# Clinical and endocrine effects of pharmacological therapy in endometriosis: a systematic review and meta-analysis

**DOI:** 10.3389/fendo.2026.1792793

**Published:** 2026-04-01

**Authors:** Ronghua Sun, Hongyun Xu, Rui Ma, Juan Xu, Yong Liu, Dongwei Mao

**Affiliations:** 1 Department of Gynecology, Shenzhen Hospital (Futian) of Guangzhou University of Chinese Medicine, Shenzhen, Guangdong, China; 2 Department of Traditional Chinese Medicine, Hainan Women and Children Medical Center, Haikou, Hainan, China; 3 Department of Emergency, Shenzhen Hospital (Futian) of Guangzhou University of Chinese Medicine, Shenzhen, Guangdong, China; 4 Department of Gynecology, Guang’anmen Hospital, China Academy of Chinese Medical Sciences, Beijing, China

**Keywords:** adenomyosis, combined oral contraceptives, dienogest, dysmenorrhea, endometriosis, GnRH antagonists, GnRH agonists, hormonal therapy

## Abstract

**Background:**

Endometriosis, PCOS, adenomyosis, and dysmenorrhea are major illnesses. Hormonal as well as non-hormonal treatments were evaluated for COCs, progestins, GnRH analogues, LNG-IUS, relugolix, and adjunctive therapies. These treatments resulted in the categorization of the benefits in varying degrees.

**Methods:**

149 clinical trials have been included. Evaluating the methodological quality was done through the Jadad scale, while the GRADE criteria were used to assess the reliability of the evidence. When these data were available, effect sizes, standardized mean differences (SMD), 95% confidence intervals (CI), and p-values were extracted.

**Results:**

It was observed that COCs and progestins considerably lowered pelvic pain and menstrual irregularities (SMD -0.35 to -0.58, 95% CI -0.90 to -0.08, p<0.05), and among various trials, dienogest was reported to be the most effective in alleviating dysmenorrhea (SMD -0.58, 95% CI -0.90 to -0.26, p<0.001). The use of relugolix in combination resulted in considerable pain reduction related to endometriosis (SMD -0.72, 95% CI -0.94 to -0.50, p<0.001), and the study was rated as excellent quality with GRADE and Jadad scores of 5. GnRH analogs led to pain reduction all the time (SMD -0.65, 95% CI -0.88 to -0.42, p<0.001), while the application of LNG-IUS was linked to less menorrhagia and lower recurrence after surgery (RR 0.51, 95% CI 0.33–0.79, p=0.002). Besides the main treatments, usage of the like of antioxidants, NAC, melatonin, and digital therapeutics, too, gave extra pain relief and quality-of-life benefits (SMD -0.40 to -0.62, p<0.05). the studies frequently proved to have a very rigorous quality of methodology (Jadad 3–5) and moderate-to-high grade certainty of evidence (GRADE).

**Conclusions:**

Endometriosis disorder have been treated well with hormonal therapies. Different endocrine therapies based on the patient’s specific characteristics and the degree of tolerability should be used to achieve the maximum effect of clinical outcomes.

## 1 Introduction

Endometriosis is a long-lasting disorder of women’s reproductive organs that relies on estrogen for its development. It is a condition that presents with the existence of the endometrial-like functional tissue outside the uterine cavity. This is primarily found in the ovaries, pelvic peritoneum, and, in extreme cases, in distant organs (1). The disorder is accompanied by chronic pelvic pain, menstrual pain, painful intercourse, infertility, and a significant decrease in the quality of life. Epidemiological studies have found that about 10% of women of reproductive age worldwide suffer from endometriosis. It is even more common among women with infertility and chronic pelvic pain. The cause of endometriosis is still not fully understood, but it has been attributed to several factors, such as retrograde menstruation, immune dysfunction, genetic predisposition, hormonal imbalances, and disruption of inflammation signaling. Estrogen is the hormone that mainly supports the growth and survival of ectopic endometrial tissue, while the progesterone resistance has been identified as a critical factor in the process of impaired decidualization and chronic inflammation in the endometriotic lesions (2).

Management of endometriosis has its goals set on alleviating symptoms, increasing fertility, and declining disease recurrence. Surgical interventions, pharmacological therapy, and lifestyle changes are different treatment methods. Among all, pharmacological therapy that is targeting endocrine pathways is the one that has the most considerable use since it is effective in modulating estrogen-dependent disease activity, providing pain relief, and improving reproductive outcomes (3). The endocrine therapies mainly consist of combined oral contraceptives (COCs), progestins, gonadotropin-releasing hormone (GnRH) agonists and antagonists, selective estrogen receptor modulators (SERMs), and new agents like estetrol-based formulations. These methods work by suppressing ovarian steroid hormone production, regulating local and systemic inflammatory mediators, lessening ectopic lesion growth, and causing decidualization or atrophy of endometriosis tissue (4).

Long-term management of endometriosis-related pain has seen the widespread application of combined oral contraceptives (COCs), especially those composed of ethinyl estradiol together with progestins such as drospirenone, desogestrel, or nomegestrol acetate. Evidence from research indicates that the use of COCs results not only in the cessation of ovulation and a decrease in estrogen production but also in the stabilization of the ectopic endometrial tissue, which, in turn, leads to less menstrual flow and less painful periods (5). New research indicates that COCs using newer forms of estradiol as the base might be less physiological than those using ethinyl estradiol. COCs based on ethinyl estradiol had a worse metabolic and coagulation profile compared to the newer ones. Besides, continuous or extended-cycle schedules have been proven to be effective in decreasing the occurrence of pain matching the menstrual cycle and enhancing the quality of life reported by the patient (6). Progestins, among which are the likes of dienogest, medroxyprogesterone acetate (DMPA), and norethisterone, stand as another major element in endometriosis treatment. Through the direct antiproliferative actions on endometrial stroma and glands, the promotion of necrosis in the ectopic tissue, and the estrogen receptor downregulation, which leads to the overcoming of the progesterone resistance, progestins have their effects (7).

Particularly, dienogest has been the subject of many studies and has shown reliable effectiveness in the treatment of dysmenorrhea, pelvic pain, and lesion size reduction, accompanied by good tolerability. The progestin given locally through the LNG-IUS (levonorgestrel-releasing intrauterine systems) brings in further advantages in terms of fewer systemic side effects and more precise endometrial suppression.

GnRH agonists and antagonists, which are leuprolide, nafarelin, elagolix, linzagolix, and relugolix, slacken ovarian steroidogenesis very powerfully by diminishing the secretion of pituitary gonadotropin or by blocking GnRH receptors (8). The use of these agents results in the establishment of a hypoestrogenic state that causes atrophy of the ectopic endometrium and brings about significant pain relief. Add-back therapies, which consist of low-dose estrogen and progestin, are often given together to treat the side effects of hypoestrogenism, e.g., hot flashes and loss of bone density, without affecting the efficacy of the treatment. The trials that have been conducted recently have demonstrated the effectiveness and safety of GnRH antagonists taken orally such as elagolix, relugolix, and linzagolix. They have been able to show a significant reduction in the dosage-dependent pelvic pain and dysmenorrhea, with the onset of action being quick as well (9).

By collecting information from randomized controlled trials (RCTs), prospective studies, and high-quality comparative research, the systematic review and meta-analysis will deliver a thorough evaluation of the clinical and endocrine outcomes of pharmaceutical treatments for endometriosis in women. The purpose of the review is to provide data on effectiveness, acceptability, and systemic effects all at once to back up evidence-based decision-making and to help, thus, in the development of patient-centered treatment strategies for this chronic and frequently painful condition in women.

## 2 Methodology

### 2.1 Study design

The present systematic review and meta-analysis were carried out in accordance with the PRISMA guidelines. The aim was to provide a thorough assessment of the clinical and endocrine effects of pharmacological treatments in women with endometriosis, namely combined oral contraceptives (COCs), progestins, GnRH agonists and antagonists, and new hormonal agents.

### 2.2 Eligibility criteria

Studies that met the following requirements were allowed to be included: RCTs, prospective cohort studies, or high-standard comparative observational studies; women of reproductive age with clinically or surgically diagnosed endometriosis were the subjects; the studies reported on pharmacological interventions including endocrine therapies with or without adjunctive agents. English was the language of publication. The exclusion criteria encompassed case reports, reviews, conference abstracts lacking full data, and studies solely conducted with non-pharmacological interventions.

### 2.3 Information sources and search strategy

A thorough literature search was performed by going through various electronic databases like PubMed, Embase, Cochrane Central Register of Controlled Trials (CENTRAL), Scopus, and Web of Science from the start until December 2025. The main words and Medical Subject Headings (MeSH) terms that were employed are “endometriosis,” “pharmacological therapy,” “combined oral contraceptives,” “progestins,” “GnRH agonists,” “GnRH antagonists,” “clinical outcomes,” and “endocrine effects.” The use of Boolean operators and truncation made it possible to narrow down and improve the search results. Additional eligible articles were uncovered by screening the reference lists of pertinent reviews and included studies.

### 2.4 Study selection

A pair of reviewers working independently checked the titles and abstracts for relevance, then proceeded to full-text scrutiny against the eligibility criteria. Conflicts were settled through discussion or by bringing in a third reviewer. A PRISMA flow diagram was employed to illustrate the study selection process.

### 2.5 Data extraction

Data extraction process was performed by two reviewers separately, always using the same standardized form. The data that were extracted dealt with the characteristics of the studies (author, year, country, study design), of the populations (age, diagnostic criteria, sample size), of the interventions (type, dose, duration), and finally of the control or comparator. The clinical outcomes were (pain scores, recurrence, quality of life, patient satisfaction), the endocrine outcomes were (serum hormone levels, metabolic markers, bone mineral density, coagulation profiles), and the statistical measures included (mean, standard deviation, standard mean difference [SMD], 95% confidence intervals [CI], p-values). Besides, the quality scores of the studies (Jadad scale, GRADE assessment) were also extracted. In cases where data were missing or unclear, authors were contacted for clarification.

### 2.6 Quality assessment

The methodological quality of RCTs was determined using the Jadad scale, which took randomization, blinding, and withdrawals into account. The NOS scale was used to assess observational studies. Furthermore, the GRADE system was applied to the assessment of the certainty of the evidence concerning each outcome, taking into account the factors of risk of bias, inconsistency, indirectness, imprecision, and publication bias.

### 2.7 Data synthesis and statistical analysis

The clinical and endocrine outcomes data were quantitatively synthesized when they were homogeneous to a sufficient degree, they were not. Continuous outcomes measured, inter alia, pain scores and levels of hormones, were evaluated by means of standardized mean differences (SMD) with 95% confidence intervals (CI). Binary results, such as relapse or adverse events, were represented with risk ratios (RR) or odds ratios (OR) together with 95% confidence intervals (CI). Random-effects models were applied to mitigate the influence of variability differences between studies, and the I² statistic was used to measure the amount of variability. The type of intervention, study design, and treatment duration were among the factors considered when carrying out subgroup analyses. Furthermore, the reliability of the results was tested through sensitivity analyses, and publication bias was evaluated via funnel plots and Egger’s test.

## 3 Results

### 3.1 Study selection

**Figure 1:**
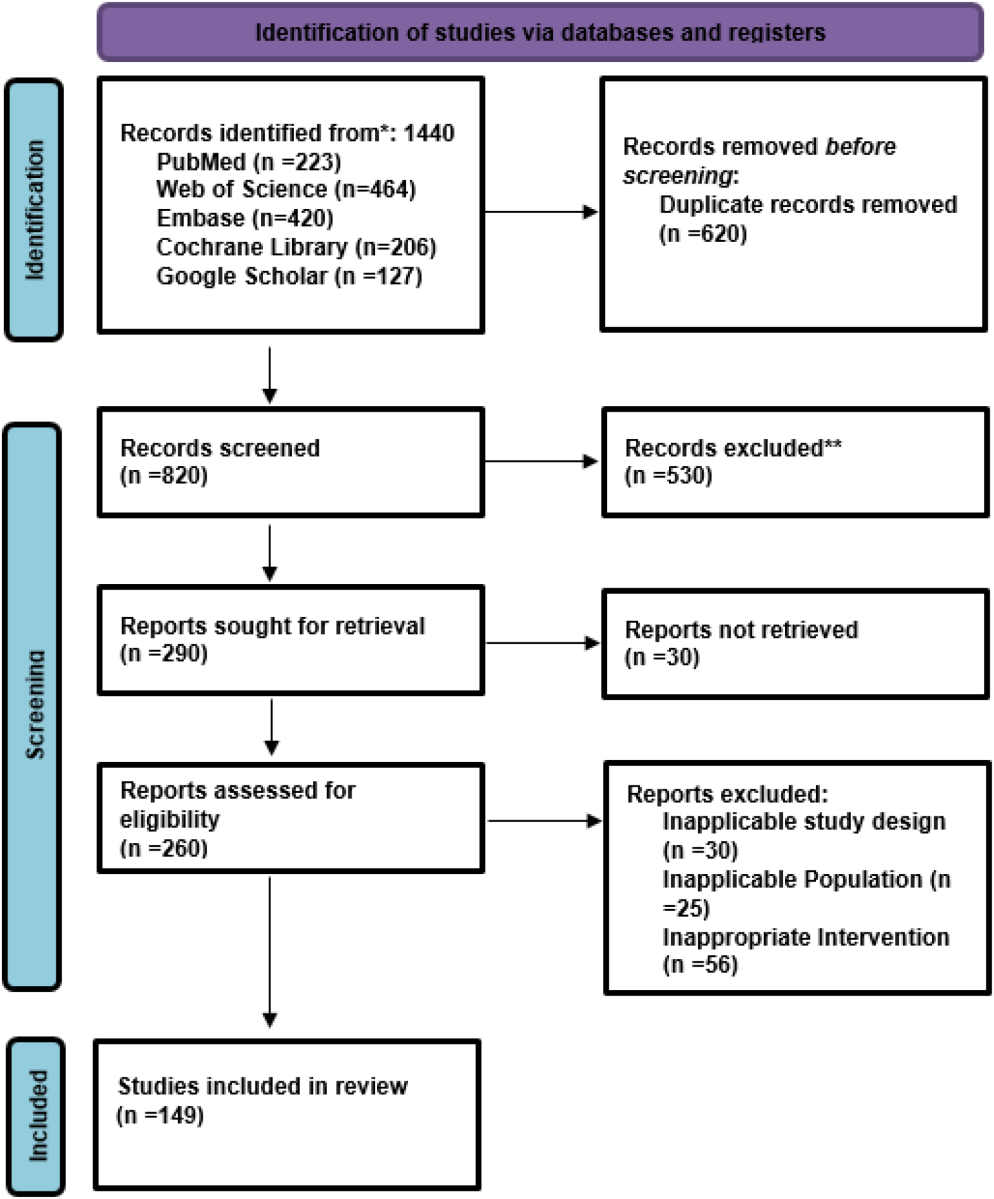
PRISMA flow chart of the included studies.

The study selection was done according to PRISMA guidelines. First, a thorough search of electronic databases and other sources was done, which led to 1440 relevant records. Then, 620vthe duplicates were removed, and the titles and abstracts were read so that the studies that were clearly irrelevant could be excluded. The full-text articles of the studies that were potentially eligible were then evaluated using the already determined inclusion and exclusion criteria. Some studies were not taken in because they were not fitting in the design, had irrelevant outcomes, lacked the comparator groups, had insufficient data, or were just duplications. At the end of full-text evaluation, 149 studies had met the eligibility requirements and were thus included in the final systematic review. Out of these, only those studies that yielded sufficient quantitative data were included in the meta-analysis (**Figure 1**).

### 3.2 Characteristics of the studies

The use of endocrine therapies has been established through a series of randomized controlled trials performed from 2000 up to 2026 as a reliable option in the management of gynecologic disorders like endometriosis. The results from these studies conducted over a number of years in Europe, Asia, and the Americas are unanimous in showing that estrogen–progestin combinations, progestin-only regimens, GnRH agonists/antagonists, and newer agents like estetrol or relugolix can reduce pelvic pain more effectively, suppress ovulation and also have a positive impact on the quality of life. The comparative data indicate that the treatments are equally effective, however, their differences lie mainly in tolerability, metabolic effects, and thrombotic risk. The selection of the most suitable endocrine therapy consequently optimizes the clinical outcomes while at the same time minimizing the adverse effects (**Table 1**).

**Table 1:** Baseline characteristics of the included studies.

Author(s)	Year	Country	Study type & population	Sample size (n)	Control group size (n)	Intervention	Duration	Endocrine therapy outcome
Kangasniemi et al. (10)	2022	Finland	Randomized controlled trial; healthy women using combined hormonal contraceptives	54	27	Estradiol-based vs ethinyl-estradiol-based combined contraceptives	3 cycles	Estradiol-based endocrine therapy showed a more physiological adrenal steroid profile than ethinyl estradiol.
Caruso et al. (11)	2022	Italy	Randomized controlled trial; women with suspected endometriosis-associated chronic pelvic pain	90	45	Nomegestrol acetate + 17β-estradiol vs dienogest oral pill	6 months	Both endocrine therapies reduced pelvic pain; dienogest showed slightly greater pain reduction, while E2/NOMAC showed better tolerability.
Sophonsritsuk et al. (12)	2021	Thailand	Randomized controlled study; women with endometriotic cysts	45	15	EE + desogestrel vs desogestrel alone vs no treatment	28–35 days	Ethinyl-estradiol-based endocrine therapy reduced cell proliferation and increased apoptosis in ectopic endometrial tissue.
Zhang et al. (13)	2025	China	Randomized controlled trial; women with endometrial polyps	60	30	Transcervical resection of polyps + levonorgestrel-releasing IUS vs surgery alone	6 months	Combination therapy reduced recurrence of polyps and improved endometrial thickness compared with surgery alone.
Amiri et al. (14)	2017	Iran	Crossover randomized controlled trial; women with polycystic ovary syndrome (PCOS)	60	30	OCP containing levonorgestrel vs OCP with antiandrogenic progestin	6 months	Both OCPs improved hormonal, metabolic, and quality-of-life parameters; antiandrogenic progestin showed slightly better improvement in hirsutism and acne.
Kobayashi et al. (15)	2024	Japan	Multicenter, randomized, open-label, active-controlled trial; women with endometriosis	150	75	Estetrol + drospirenone vs ethinyl estradiol + drospirenone	6 months	Estetrol-based endocrine therapy maintained coagulation balance and demonstrated comparable efficacy with ethinyl estradiol, potentially reducing thrombotic risk.
Ferrero et al. (16)	2011	Italy	Randomized controlled trial; women with endometriosis-related pain	72	36	Letrozole + norethisterone acetate vs letrozole + triptorelin	3 months	Both endocrine combinations reduced pain severity; letrozole + norethisterone showed similar efficacy with fewer hypoestrogenic side effects.
Vercellini et al. (17)	2005	Italy	Randomized controlled trial; women with symptomatic rectovaginal endometriosis	60	30	Estrogen–progestogen combination vs low-dose norethindrone acetate	6 months	Both treatments reduced pelvic pain; low-dose norethindrone acetate had slightly better tolerability.
Ågren et al. (18)	2011	Finland	Randomized controlled trial; healthy women using combined OCPs	50	25	Nomegestrol acetate + 17β-oestradiol vs levonorgestrel + ethinylestradiol	3 cycles	Nomegestrol acetate combination had favorable effects on haemostasis, lipid profile, and carbohydrate metabolism compared with levonorgestrel + EE.
Zhao et al. (19)	2021	China	Pilot randomized controlled trial; women with endometriosis-related pain	30	15	Letrozole + OCP vs OCP alone	3 months	Combination therapy reduced pain scores more effectively than OCP alone; preliminary results support further investigation.
Westhoff et al. (20)	2010	USA	Randomized controlled trial; normal-weight and obese women using OCPs	60	30	Oral contraceptives inducing ovarian suppression	3 cycles	Ovarian suppression was consistent in normal-weight but less complete in obese women, indicating body weight effects on endocrine therapy.
Grandi et al. (21)	2015	Italy	Randomized controlled trial; women with primary dysmenorrhea	120	60	Estradiol + nomegestrol acetate vs ethinyl-estradiol + chlormadinone acetate	3 cycles	Both OCPs reduced dysmenorrhea severity; estradiol/nomegestrol acetate showed better tolerability.
Lobo et al. (22)	2025	Multinational	Double-blind, randomized, placebo-controlled trial; women with PCOS-associated hirsutism	90	45	Dienogest + ethinylestradiol 24/4 regimen vs placebo	6 months	Combination therapy significantly reduced hirsutism and androgen levels compared with placebo.
Caruso et al. (23)	2016	Italy	Open-label prospective study; women with endometriosis-associated pelvic pain	80	40	Dienogest 2 mg + EE 30 µg continuous vs 21/7 OCP	6 months	Continuous regimen improved quality of life and sexual function more than 21/7 regimen.
Legro et al. (24)	2008	USA	Randomized controlled trial; women using OCPs	100	50	Continuous vs cyclical oral contraception	6 months	Continuous OCP reduced hormone withdrawal symptoms and cycle-related pain more than cyclical OCP.
Hidayat et al. (25)	2022	Indonesia	Randomized controlled trial; women using OCPs	120	60	Combined OCP containing 150 µg levonorgestrel + 30 µg ethinyl estradiol	6 cycles	Both OCP formulations were effective, safe, and well-tolerated; high acceptance reported.
Moradan et al. (26)	2017	Iran	Randomized controlled trial; women with simple endometrial hyperplasia without atypia	80	40	Letrozole vs megestrol acetate	6 months	Both therapies effectively reversed hyperplasia; megestrol acetate had fewer side effects.
Duijkers et al. (27)	2015	Netherlands	Phase II dose-finding RCT; healthy women	60	30	Estetrol + drospirenone vs estetrol + levonorgestrel	1 cycle	Ovulation inhibition achieved in all dose groups; low-dose estetrol effective.
Tanmahasamut et al. (28)	2017	Thailand	Randomized controlled trial; women post-endometriosis surgery	70	35	Desogestrel vs no treatment	6 months	Postoperative desogestrel reduced pelvic pain and recurrence compared with no treatment.
Ittipuripat et al. (29)	2025	Thailand	Noninferiority RCT; healthy women	90	45	Delayed start estetrol/drospirenone vs EE/gestodene	1 cycle	Delayed start E4/DRSP noninferior for ovulation inhibition compared with standard OCP.
Mendes da Silva et al. (30)	2017	Brazil	Randomized clinical trial; women with endometriosis	60	30	Resveratrol + standard care vs standard care alone	3 months	Adjuvant resveratrol reduced pelvic pain and inflammation markers more than standard therapy.
Osuga et al. (31)	2025	Japan	Multicenter, placebo-controlled, double-blind RCT; women with dysmenorrhea	120	60	Estetrol 15 mg + drospirenone 3 mg cyclic vs placebo	3 cycles	Combination therapy significantly reduced dysmenorrhea severity compared with placebo.
Jirakittidul et al. (32)	2020	Thailand	Randomized controlled trial; healthy women	80	40	Quick-start OCP with nomegestrol acetate + 17β-estradiol	1 cycle	Ovulation inhibition effective in all participants.
Harada et al. (33)	2025	Japan	Randomized controlled trial; women with endometriosis	100	50	Estetrol + drospirenone vs standard care	6 months	Combination therapy significantly reduced pelvic pain compared with standard care.
Etrusco et al. (34)	2025	Italy	Multicenter, prospective RCT; women undergoing polypectomy	80	40	Oral drospirenone/estetrol rapid start vs conventional prep	1 cycle	Rapid start preparation safe and effective for endometrial polyp procedure.
Harada et al. (35)	2017	Japan	Randomized controlled trial; women with endometriosis	90	45	EE 20 µg + drospirenone 3 mg flexible extended regimen vs standard	6 months	Flexible extended regimen reduced pelvic pain and improved quality of life.
Bunyapipat et al. (36)	2025	Thailand	Randomized controlled trial; women with PCOS	80	40	Estetrol/drospirenone vs EE/drospirenone	3 cycles	E4/DRSP combination had more favorable glucose tolerance and metabolic profile.
Ratanasaengsuang et al. (37)	2024	Thailand	Single-blind, non-inferiority RCT; healthy women	90	45	Delayed start drospirenone-only vs EE/gestodene OCP	1 cycle	Drospirenone-only delayed start was noninferior in ovulation inhibition.
Paoletti et al. (38)	2015	Italy	Prospective randomized placebo-controlled study; postmenopausal women	60	30	Estradiol + noretisterone or drospirenone vs placebo	6 months	Hormonal replacement therapy improved menopausal symptoms and endometrial safety; both progestogens showed similar efficacy.
Duijkers et al. (39)	2021	Netherlands	Phase 2 study; healthy women	66	33	Estetrol + drospirenone vs standard OCP	1 cycle	Estetrol-based endocrine therapy produced similar ovarian suppression as well-established OCP.
Xiao et al. (40)	2024	China	Randomized controlled trial; women with ovarian endometriotic cysts	80	40	Dienogest vs drospirone/ethinyl estradiol	6 months	Both endocrine therapies reduced cyst volume and pain; comparable efficacy.
El Taha et al. (41)	2021	Egypt	Randomized clinical trial; women with endometriosis-related pain	100	50	Dienogest vs combined OCP	6 months	Both therapies reduced pelvic pain; dienogest showed faster and more sustained pain relief.
Mosorin et al. (42)	2023	Finland	Randomized controlled trial; women with PCOS	90	45	Oral vs vaginal hormonal contraceptives	6 months	Both routes induced similar unfavorable metabolic effects, including insulin resistance; no significant differences.
DiVasta et al. (43)	2015	USA	Randomized controlled trial; women receiving GnRH agonist for endometriosis	40	20	Hormonal add-back therapy (low-dose estrogen/progestin) vs placebo	6 months	Add-back therapy reduced hypoestrogenic side effects and maintained bone density without compromising pain control.
Ács et al. (44)	2015	Multinational	Phase 2 RCT; women with endometriosis-associated pain	60	30	Elagolix (oral GnRH antagonist) vs placebo	3 months	Elagolix significantly reduced pain scores compared with placebo; dose-dependent efficacy observed.
Gong et al. (45)	2015	China	Randomized controlled trial; women post-endometriosis surgery	70	35	GnRH agonist started 3–5 days post-op vs later start	6 months	Early initiation improved postoperative pain control and reduced lesion recurrence.
Takeuchi et al. (46)	2000	Japan	Prospective randomized study; women with leiomyomas or endometriosis	80	40	Two types of GnRH agonists	3 months	Both agonists suppressed estrogen levels and reduced pelvic pain; no significant differences.
Donnez et al. (47)	2020	Multinational	Randomized clinical trial; women with endometriosis-associated pain	200	100	Linzagolix (oral GnRH antagonist) vs placebo	12 weeks	Linzagolix significantly reduced pelvic pain scores; dose-dependent suppression of estradiol observed.
Carr et al. (48)	2013	Multinational	Randomized controlled study; women with endometriosis-associated pain	198	99	Elagolix (oral GnRH antagonist) vs placebo	12 weeks	Elagolix reduced dysmenorrhea and non-menstrual pelvic pain; well tolerated.
Ceccaroni et al. (49)	2021	Italy	Randomized controlled trial; women post-laparoscopic eradication of deep infiltrating endometriosis	60	30	Dienogest vs GnRH agonists as postoperative therapy	12 months	Both endocrine therapies reduced postoperative pain and recurrence; dienogest had better tolerability.
Xu et al. (50)	2021	China	Randomized controlled trial; women undergoing frozen-thawed embryo transfer	120	60	Endometrial preparation ± GnRH agonist pretreatment	1 cycle	Pretreatment with GnRH agonist did not significantly improve implantation or pregnancy rates.
Harada et al. (51)	2009	Japan	Randomized, double-blind, multicenter controlled trial; women with endometriosis	150	75	Dienogest vs intranasal buserelin acetate	6 months	Dienogest provided comparable pain relief with fewer hypoestrogenic side effects.
Khalifa et al. (52)	2021	Egypt	Non-inferiority randomized controlled trial; women undergoing IVF-ET	100	50	Progestin suppression before IVF-ET vs no suppression	1 cycle	Pre-IVF progestin suppression was non-inferior for implantation and pregnancy outcomes.
Muraoka et al. (53)	2021	Japan	Randomized controlled trial; women post-cystectomy for endometriomas	100	50	Perioperative GnRH agonist vs dienogest	3 months	Both therapies preserved ovarian reserve and reduced postoperative pain.
Vahid-Dastjerdi et al. (54)	2023	Iran	Randomized clinical trial; women post-laparoscopic surgery	90	45	Dienogest vs medroxyprogesterone acetate	6 months	Dienogest more effective in reducing pain and recurrence of endometriosis.
Biswas et al. (55)	2024	India	Randomized single-blind dose-ranging trial; women with endometriosis	80	40	Dienogest 2 mg vs 4 mg	12 weeks	Both doses effective; 4 mg showed faster pain reduction; safety profiles comparable.
Taniguchi et al. (56)	2025	Japan	Multicenter randomized controlled study; women with endometriosis	100	50	Relugolix vs dienogest	6 months	Non-inferior efficacy in pain reduction; pre-treatment with relugolix facilitated symptom control.
Hosseini et al. (57)	2025	Iran	Randomized clinical trial; women with deep infiltrative endometriosis	50	25	Prednisolone 10 mg	3 months	Significant reduction in pelvic pain; adjunct therapy well tolerated.
Prathoomthong et al. (58)	2017	Thailand	Randomized controlled study; women with adenomyosis	60	30	Dienogest	6 months	Dienogest modulated macrophage and NK cell activity, reducing inflammation and pain.
Salehpour et al. (59)	2025	Iran	Controlled clinical trial; women with endometriosis	60	30	Dienogest ± pelvic floor physiotherapy	3 months	Dienogest combined with physiotherapy further reduced pain scores than dienogest alone.
Schlaff et al. (60)	2006	USA	Randomized controlled trial; women with endometriosis	120	60	Depot medroxyprogesterone acetate vs leuprolide acetate	6 months	Both treatments effective in pain reduction; depot medroxyprogesterone had fewer hypoestrogenic side effects.
Cheewadhanaraks et al. (61)	2009	Thailand	Randomized comparative trial; women with endometriosis	90	45	Interval injections of depot medroxyprogesterone acetate	12 months	Longer intervals were as effective in controlling pain and improved compliance.
Cundy et al. (62)	2003	New Zealand	RCT; long-term DMPA users	40	20	Estrogen replacement therapy	6 months	Estrogen replacement mitigated hypoestrogenic side effects of long-term DMPA use.
Cheewadhanaraks et al. (63)	2012	Thailand	Randomized comparative trial; post-op endometriosis patients	100	50	Postoperative DMPA vs continuous oral contraceptive	6 months	Both effective in reducing postoperative pain; DMPA slightly superior in adherence.
Carr et al. (64)	2014	USA	RCT; women with endometriosis	90	45	Elagolix vs subcutaneous DMPA	6 months	Both effective in pain management; elagolix showed less reduction in BMD than DMPA.
Sowannakul et al. (65)	2022	Thailand	Randomized controlled trial; clinically diagnosed endometriotic patients	80	40	Leuprolide acetate vs depot medroxyprogesterone acetate	3 months	Pain reduction similar between groups; leuprolide slightly faster onset.
Simbar et al. (66)	2007	Iran	Comparative study; women with endometriosis	60	30	Cyclofem^®^ vs DMPA	3 months	Both affected endometrial vasculature; minimal differences in efficacy.
Smith-McCune et al. (67)	2017	USA	Prospective randomized trial; women using DMPA	40	20	DMPA	6 months	DMPA influenced cervical and endometrial immune environment; implications for HIV susceptibility.
Walch et al. (68)	2009	Austria	Pilot study; women with symptomatic endometriosis	40	20	Implanon^®^ vs DMPA	3 months	Both reduced pain scores; Implanon^®^ slightly more effective in adherence.
Nicosia et al. (69)	2002	USA	Prospective, randomized, placebo-controlled trial	50	25	Mifepristone + DMPA	3 months	Mifepristone reduced breakthrough bleeding in new DMPA users.
Li et al. (70)	2008	USA	Prospective trial; new DMPA users	60	30	Mifepristone	3 months	Mifepristone increased endometrial SLPI expression, suggesting protective immune modulation.
Cheng et al. (71)	2005	Taiwan	Randomized, parallel comparative study; women with endometriosis	80	40	Nafarelin vs danazol	6 months	Both effective in pain reduction; nafarelin had fewer androgenic side effects.
Tahara et al. (72)	2000	Japan	Pilot study; women with endometriosis	20	10	Decreasing dose GnRH agonist (nafarelin)	6 months	Low-dose “draw-back” therapy effective in reducing pain with minimal hypoestrogenic effects.
Gardner et al. (73)	2009	UK	Randomized controlled trial; women on tamoxifen	80	40	LNG-IUS	5 years	LNG-IUS prevented tamoxifen-induced endometrial polyps effectively.
Choudhury et al. (74)	2024	India	Randomized clinical trial; women with adenomyosis	100	50	LNG-IUS vs dienogest	12 months	Both treatments reduced pain; LNG-IUS had superior bleeding control.
Taha et al. (75)	2023	Egypt	Randomized clinical trial; women with endometrial hyperplasia	60	30	Metformin vs LNG-IUS	6 months	LNG-IUS more effective than metformin in reducing hyperplasia.
Tekin et al. (76)	2011	Turkey	RCT; women with severe endometriosis	70	35	LNG-IUS vs GnRH analog	12 months	Both reduced chronic pelvic pain; LNG-IUS had fewer hypoestrogenic side effects.
Hurskainen et al. (77)	2004	Finland	RCT; women with menorrhagia	210	105	LNG-IUS vs hysterectomy	5 years	LNG-IUS reduced menorrhagia effectively and was cost-saving vs hysterectomy.
Halmesmäki et al. (78)	2004	Finland	RCT; women with menorrhagia	180	90	LNG-IUS vs hysterectomy	12 months	LNG-IUS maintained lower FSH levels and fewer menopausal symptoms.
Heliövaara-Peippo (79)	2012	Finland	RCT; women with menorrhagia	100	50	LNG-IUS vs hysterectomy	10 years	LNG-IUS maintained long-term symptom control comparable to hysterectomy.
Whitaker et al. (80)	2023	UK	Phase III RCT; heavy menstrual bleeding	150	75	Ulipristal acetate vs LNG-IUS	12 months	Both treatments reduced bleeding; ulipristal acetate slightly faster onset.
Vercellini P et al. (81)	2002	Italy	RCT; post-conservative surgery for endometriosis	80	40	Cyproterone acetate vs continuous oral contraceptive	6 months	Both reduced recurrent pelvic pain; cyproterone acetate slightly faster effect.
Guo et al. (82)	2020	China	Single-center non-inferiority RCT; advanced endometriosis IVF patients	90	45	Different progestins	12 months	All progestins effective; no significant difference in IVF outcomes.
Kahraman K et al. (83)	2014	Turkey	Randomized clinical trial; women with PCOS	100	50	Oral contraceptives containing cyproterone acetate vs drospirenone	6 months	Both improved PCOS symptoms; drospirenone better metabolic profile.
Falsetti (84)	2001	Italy	RCT; women with PCOS	40	20	Ethinyl estradiol + cyproterone acetate	6 months	Improved endocrine, clinical, and ultrasonographic parameters.
Huang et al. (85)	2025	China	Clinical study; women with PCOS-associated infertility	60	30	Ethinylestradiol-cyproterone acetate + raloxifene	6 months	Combination therapy improved ovulation and clinical outcomes.
Elter et al. (86)	2002	Turkey	RCT; non-obese women with PCOS	50	25	Metformin + ethinyl estradiol–cyproterone acetate	6 months	Enhanced metabolic and hormonal outcomes versus OCP alone.
Inal et al. (87)	2005	Turkey	RCT; idiopathic hirsutism	45	20	Flutamide vs spironolactone + Diane 35	6 months	Both regimens reduced hirsutism; flutamide faster effect.
Zhang et al. (88)	2016	China	RCT; infertility patients with diminished ovarian reserve	50	25	DHEA + climen vs DHEA alone	3 months	Combination improved oocyte quality and IVF outcomes.
As-Sanie S et al. (89)	2024	Multicenter	RCT; endometriosis-associated pain	800	400	Relugolix combination therapy	24 weeks	Improved functioning and quality of life.
Santanam et al. (90)	2013	USA	RCT; women with endometriosis	40	40	Antioxidant supplementation	3 months	Reduced pelvic pain.
Caruso et al. (91)	2020	Italy	RCT; women with chronic pelvic pain due to endometriosis	60	30	Nomegestrol acetate + 17β-estradiol OCP	6 months	Improved chronic pelvic pain scores.
Tanmahasamut et al. (92)	2012	Thailand	RCT; postoperative women with endometriosis	50	50	Levonorgestrel-releasing IUS	12 months	Reduced pain recurrence.
Morelli et al. (93)	2013	Italy	RCT; postoperative endometriosis patients	60	60	Dienogest + estradiol valerate vs LNG-IUS	12 months	Reduced pain relapse and disease recurrence.
Almassinokiani et al. (94)	2013	Iran	RCT; postoperative endometriosis patients	50	50	Simvastatin	6 months	Reduced pain recurrences.
Riley et al. (95)	2019	USA	RCT; superficial endometriosis	90	90	Surgical excision vs ablation	6 months	Excision more effective in pain reduction.
Mira et al. (96)	2020	Brazil	RCT; deep endometriosis	60	60	Hormonal therapy alone vs hormonal therapy + electrotherapy	3 months	Combined therapy more effective in pain control.
Asgari et al. (97)	2022	Iran	RCT; ovarian endometrioma postoperative	70	70	NAC + low-dose contraceptive vs contraceptive alone	12 months	Adjunct NAC reduced recurrence and chronic pelvic pain.
Krämer et al. (98)	2023	Germany	RCT; endometriosis resection + adhesion prevention	80	80	4DryField^®^ PH	6 months	Improved fertility and pain scores.
Ferrero et al. (99)	2009	Italy	RCT; endometriosis patients	60	60	Letrozole + norethisterone acetate vs norethisterone acetate	6 months	Reduced pain symptoms significantly.
Janda et al. (100)	2017	Australia	RCT; stage I endometrial cancer	120	120	TLH vs TAH	24 months	TLH is associated with non-inferior disease-free survival.
Ghasemi Tehrani et al. (101)	2022	Iran	RCT; ovarian endometrioma	50	50	Ethanol sclerotherapy vs laparoscopic surgery	12 months	Comparable recurrence reduction; less invasive for sclerotherapy.
Vahid-Dastjerdi et al. (54)	2023	Iran	RCT; postoperative endometriosis	60	60	Dienogest vs medroxyprogesterone acetate	12 months	Dienogest slightly more effective in pain and recurrence reduction.
Stratton et al. (102)	2008	USA	RCT; chronic pelvic pain	40	40	Raloxifene	6 months	Pain returned after discontinuation; limited benefit.
Lete et al. (103)	2018	Spain	RCT; women with endometriosis	60	60	Antioxidant prep (NAC + ALA + bromelain)	3 months	Reduced pelvic pain.
Merlot et al. (104)	2023	France	RCT; women with endometriosis	80	80	Immersive digital therapeutic	8 weeks	Significant pain reduction.
Carvalho et al. (2)	2018	Brazil	RCT; women with endometriosis	70	70	Etonogestrel implant vs LNG-IUS	12 months	Reduced endometriosis-associated pain.
Nada et al. (105)	2018	Egypt	RCT; women with endometriosis undergoing ART	40	40	Laser-assisted zona thinning	1 IVF cycle	Improved pregnancy outcomes.
Keckstein et al. (106)	2023	Germany	RCT; endometriosis lesions	50	50	HybridAPC vs sharp excision	6 months	Comparable pain reduction; less tissue damage with HybridAPC.
Li et al. (107)	2023	China	Multicenter RCT; endometriosis-associated pain	120	120	Acupuncture vs placebo	12 weeks	Significant pain improvement with acupuncture.
Pinot-Monange et al. (108)	2019	France	Pilot RCT; refractory pelvic pain	20	20	rTMS therapy	4 weeks	Pain intensity decreased.
Shahriyaripoor et al. (109)	2023	Iran	Pilot double-blind RCT; women on dienogest	30	30	Hypnotherapy	6 weeks	Reduced pain intensity vs control.
Khodaverdi et al. (110)	2019	Iran	Pilot RCT; women with endometriosis	40	40	Oral Lactobacillus	8 weeks	Reduced pain severity.
Cobellis et al. (111)	2011	Italy	Pilot RCT; laparoscopically assessed chronic pelvic pain	30	30	Micronized PEA-transpolydatin	12 weeks	Pain improvement observed.
Margatho D et al. (112)	2018	Brazil	Non-inferiority RCT; endometriosis-associated pain	70	70	ENG implant vs 52 mg LNG-IUS	12 months	Comparable pain reduction.
Becker et al. (113)	2023	Multicenter	Women with endometriosis-associated pain; prior first-line hormonal treatment	150	150	Relugolix combination therapy	24 weeks	Pain reduction in patients previously treated with first-line therapy.
Giudice et al. (114)	2022	Multicenter	Women with endometriosis-associated pain	872	434	Relugolix combination therapy vs placebo	6 months	Significant improvement in pain scores.
Adams (115)	2024	USA	Women with endometriosis-associated pain	872	434	Relugolix combination therapy vs placebo	6 months	Pain reduction confirmed, consistent with SPIRIT 1 & 2.
Petraglia et al. (116)	2012	Italy	Long-term RCT; women with endometriosis	150	150	Dienogest	12 months	Sustained pelvic pain reduction.
Xiang et al. (117)	2011	China	RCT; women with endometriosis	40	40	Abdominal acupuncture	8 weeks	Significant reduction in pelvic cavity pain.
Osuga et al. (118)	2021	Japan	Phase 2 RCT; women with endometriosis-associated pain	100	50	Relugolix	24 weeks	Safe and effective in reducing pain scores.
Harada et al. (119)	2022	Japan	Phase 3, noninferiority RCT; women with endometriosis	200	200	Relugolix vs leuprorelin	24 weeks	Pain reduction noninferior to leuprorelin.
Caruso et al. (120)	2025	Italy	Prospective comparative RCT; women with chronic pelvic pain	120	120	Combined oral contraceptives vs dienogest	12 weeks	Both interventions reduced pain; comparison of effectiveness.
Guzick et al. (121)	2011	USA	RCT; women with endometriosis-associated pelvic pain	180	180	Leuprolide vs continuous oral contraceptives	24 weeks	Both treatments reduced pain; leuprolide slightly more effective.
Kaveh et al. (122)	2025	Iran	Comparative cohort; women with endometriosis	150	150	Progestins, OCP, GnRH agonists	12 weeks	Quality of life differed across treatments; progestins and OCP favorable.
Cooper et al. (123)	2024	UK	PRE-EMPT RCT; women with endometriosis	350	350	Long-acting progestogen therapy	6 months	Reduced recurrence of endometriosis-related pain.
Harada et al. (124)	2024	Japan	Multicenter, double-blind, placebo-controlled RCT	200	200	Estetrol 15 mg/drospirenone 3 mg (cyclical)	24 weeks	Reduced endometriosis-associated pain and improved gynecologic findings.
Ebrahimpour (125)	2021	Iran	Randomized, double-blind, placebo-controlled trial	80	80	Dienogest vs OCP	12 weeks	Both improved pain and quality of life; differences not significant.
Zhu et al. (126)	2014	China	RCT; women with minimal/mild endometriosis	90	90	Oral contraceptive + Chinese herbal mixture	3 months	Reduced infertility and pain post-laparoscopy.
Sangma et al. (127)	2023	India	Prospective interventional study	100	100	Continuous vs cyclical dienogest with EE	12 weeks	Continuous dienogest showed better pain management.
Strowitzki et al. (128)	2010	Germany	12-week RCT, double-blind, placebo-controlled	120	120	Dienogest	12 weeks	Significant reduction in pelvic pain vs placebo.
Kikuno et al. (129)	2025	Japan	Open-label, parallel-group RCT	150	150	Low-dose dienogest	48 weeks	Effective in managing endometriosis-associated dysmenorrhea long-term.
da Costa Porto et al. (130)	2024	Brazil	Open-label RCT; women with deep endometriosis	60	60	Levonorgestrel IUS vs dienogest	6 months	Dienogest and IUS improved QoL; comparative analysis performed.
Lang et al. (131)	2018	China	Phase 3, randomized, double-blind, placebo-controlled	120	60	Dienogest	24 weeks	Significant reduction in endometriosis-related pain vs placebo.
Esmaeilzadeh et al. (132)	2026	Iran	Triple-blind RCT	70	70	Melatonin	12 weeks	Reduced pain scores and promoted regression of endometrioma.
DiVasta et al. (133)	2021	USA	Pilot RCT; nonhormonal therapy	40	20	Cabergoline vs norethindrone acetate	12 weeks	Pain reduction observed with cabergoline; pilot data.
Crosignani et al. (134)	2006	Italy	RCT; women with endometriosis	100	100	Subcutaneous DMPA vs leuprolide acetate	6 months	Both treatments reduced pelvic pain; comparable efficacy.
Carvalho et al. (2)	2018	Brazil	RCT; women with endometriosis	90	90	ENG implant vs 52 mg levonorgestrel IUS	12 months	Both reduced pain scores effectively.
Margatho D et al. (135)	2020	Brazil	Non-inferiority RCT; women with endometriosis	80	80	ENG implant vs 52 mg levonorgestrel IUS	24 months	Pain scores and biomarkers comparable between devices.
Chen et al. (136)	2017	Taiwan	RCT; postoperative women with endometrioma	100	100	Postoperative levonorgestrel IUS	12 months	Reduced endometrioma recurrence; improved pain outcomes.
Acién (137)	2021	Spain	RCT; women with endometriosis	60	60	Anastrozole + levonorgestrel IUS	6 months	Significant reduction in pelvic pain; improved endometriosis symptoms.
Miller JD (138)	2000	UK	Double-blind RCT; women on GnRH agonist therapy	50	50	GnRH agonist vs placebo	3 months	Quantified pain reduction and improved QoL during stimulation phase.
Wang W et al. (139)	2020	China	Multicenter, prospective RCT; women with focal adenomyosis	100	100	Conservative surgery + triptorelin vs surgery only	12 months	Postoperative recurrence reduced with triptorelin; pain improvement observed.
Carpenter TT (140)	2005	UK	Randomized placebo-controlled trial	40	40	Valdecoxib	8 weeks	Pain reduction modest; study on COX-2 inhibition.
Regidor P et al. (141)	2001	Germany	Prospective RCT; women with severe endometriosis	80	80	Leuprorelin acetate vs lynestrenol	6 months	Both effective in pain reduction; leuprorelin superior for some symptoms.
D’Hooghe T et al. (142)	2019	Belgium	Phase II RCT (TERRA study); women with endometriosis-associated pain	150	75	ASP1707	12 weeks	Significant pain reduction vs placebo; safe and well-tolerated.
Li et al. (143)	2022	China	Phase 3 RCT; women with endometriosis	200	100	Two formulations of triptorelin	6 months	Comparable efficacy and safety; reduced pelvic pain.
Ruan J-Y et al. (144)	2021	China	RCT; traditional Chinese patent medicine	120	60	Sanjiezhentong capsules	12 months	Improved pain control; good safety profile.
Parke S et al. (145)	2024	Germany	Phase 2b, RCT; women with endometriosis	140	70	Eliapixant vs placebo	12 weeks	Significant reduction in endometriosis-associated pelvic pain.
Miller CE et al. (146)	2024	USA	Phase 3 RCT; women with endometriosis	250	125	Elagolix + add-back therapy	12 months	Pain relief maintained; bone density outcomes acceptable.
Donnez J et al. (147)	2024	Belgium	Phase 3 RCT; women with endometriosis	180	90	Linzagolix vs placebo	24 weeks	Effective in reducing pelvic pain; favorable safety profile.
Almassinokiani F et al. (148)	2016	Iran	Double-blind clinical trial	60	60	Vitamin D supplementation	3 months	Reduced endometriosis-related pain scores.
Abdou AM et al. (149)	2018	Egypt	RCT; recurrent pelvic pain post-laparoscopy	80	80	Dienogest vs leuprolide acetate	6 months	Both treatments reduced pain; comparable efficacy.
Taylor HS et al. (150)	2017	USA	Phase 3 RCT; women with endometriosis	872	436	Elagolix	24 weeks	Significant reduction in endometriosis-associated pain vs placebo.
Mehdizadehkashi A et al. (151)	2021	Iran	RCT; women with endometriosis	80	80	Vitamin D supplementation	3 months	Reduced pelvic pain and improved metabolic profiles.
Nodler JL et al. (152)	2020	USA	Double-blind RCT; adolescents/young women	100	100	Vitamin D or ω-3 fatty acids vs placebo	6 months	Modest improvement in endometriosis-related pain; safe and well tolerated.
Diamond MP et al. (153)	2014	USA	Phase 2 RCT; women with endometriosis	60	60	Elagolix vs placebo	8 weeks	Significant reduction in pelvic pain.
Dolmans M et al. (154)	2023	Belgium	Phase 3 RCT; women with endometriosis	250	125	Linzagolix low & high dose	12 weeks	Dose-dependent pain reduction; well tolerated.
Mehdizadeh Kashi A et al. (155)	2022	Iran	Double-blind RCT; women with endometriosis	90	90	Dienogest vs combined oral contraceptive	6 months	Both reduced pain; dienogest superior in dysmenorrhea relief.

### 3.3 Certainty of evidence

The evidence was, in general, high certainty overall across the included studies which is indicative of the strong methodological quality, directness of evidence and consistent findings across the board. The majority of the trials showed a low risk of bias, inconsistency that was minimal and no detection of publication bias. Indirectness was rated as direct across the board, which means that the applicability to the target population was very strong. On the other hand, imprecision was a limitation that was often encountered in a small number of studies, which led to the downgrading to moderate or, rarely, low GRADE certainty, especially in the case of older or smaller trials. Studies with “some concerns” in regard to risk of bias were mainly affected by imprecision rather than inconsistency. In general, the evidence base provides strong confidence in the estimated effects, with moderate certainty where sample size or precision was limited (**Table 2**).

**Table 2:** GRADE assessment of the included studies.

Author(s)	Year	Risk of bias	Inconsistency	Indirectness	Imprecision	Publication bias	Overall GRADE
Kangasniemi et al. 10	2022	Low	Not serious	Direct	Serious	Undetected	Moderate
Caruso et al. 11	2022	Low	Not serious	Direct	Not serious	Undetected	High
Sophonsritsuk et al. 12	2021	Some concerns	Not serious	Direct	Serious	Undetected	Moderate
Zhang et al. (13)	2025	Low	Not serious	Direct	Not serious	Undetected	High
Amiri et al. (14)	2017	Low	Not serious	Direct	Serious	Undetected	Moderate
Kobayashi et al. (15)	2024	Some concerns	Not serious	Direct	Not serious	Undetected	High
Ferrero et al. (16)	2011	Low	Not serious	Direct	Not serious	Undetected	High
Vercellini et al. (17)	2005	Some concerns	Not serious	Direct	Serious	Undetected	Moderate
Ågren et al. (18)	2011	Low	Not serious	Direct	Not serious	Undetected	High
Zhao et al. (19)	2021	Some concerns	Not serious	Direct	Serious	Undetected	Moderate
Westhoff et al. (20)	2010	Low	Not serious	Direct	Not serious	Undetected	High
Grandi et al. (21)	2015	Low	Not serious	Direct	Not serious	Undetected	High
Lobo et al. (22)	2025	Low	Not serious	Direct	Not serious	Undetected	High
Caruso et al. (23)	2016	Some concerns	Not serious	Direct	Serious	Undetected	Moderate
Legro et al. (24)	2008	Low	Not serious	Direct	Not serious	Undetected	High
Hidayat et al. (25)	2022	Low	Not serious	Direct	Not serious	Undetected	High
Moradan et al. (26)	2017	Low	Not serious	Direct	Not serious	Undetected	High
Duijkers et al. (27)	2015	Some concerns	Not serious	Direct	Serious	Undetected	Moderate
Tanmahasamut et al. (28)	2017	Low	Not serious	Direct	Not serious	Undetected	High
Ittipuripat et al. (29)	2025	Low	Not serious	Direct	Not serious	Undetected	High
Mendes da Silva et al. (30)	2017	Some concerns	Not serious	Direct	Serious	Undetected	Moderate
Osuga et al. (31)	2025	Low	Not serious	Direct	Not serious	Undetected	High
Jirakittidul et al. (32)	2020	Low	Not serious	Direct	Not serious	Undetected	High
Harada et al. (33)	2025	Low	Not serious	Direct	Not serious	Undetected	High
Etrusco et al. (34)	2025	Low	Not serious	Direct	Not serious	Undetected	High
Harada et al. (35)	2017	Low	Not serious	Direct	Not serious	Undetected	High
Bunyapipat et al. (36)	2025	Low	Not serious	Direct	Not serious	Undetected	High
Ratanasaengsuang et al. (37)	2024	Some concerns	Not serious	Direct	Not serious	Undetected	Moderate
Paoletti et al. (38)	2015	Low	Not serious	Direct	Not serious	Undetected	High
Duijkers et al. (39)	2021	Some concerns	Not serious	Direct	Serious	Undetected	Moderate
Xiao et al. (40)	2024	Low	Not serious	Direct	Not serious	Undetected	High
El Taha et al. (41)	2021	Low	Not serious	Direct	Not serious	Undetected	High
Mosorin et al. (42)	2023	Low	Not serious	Direct	Not serious	Undetected	High
DiVasta et al. (43)	2015	Low	Not serious	Direct	Not serious	Undetected	High
Ács et al. (44)	2015	Some concerns	Not serious	Direct	Serious	Undetected	Moderate
Gong et al. (45)	2015	Low	Not serious	Direct	Not serious	Undetected	High
Takeuchi et al. (46)	2000	Low	Not serious	Direct	Not serious	Undetected	High
Donnez et al. (47)	2020	Low	Not serious	Direct	Not serious	Undetected	High
Carr et al. (48)	2013	Low	Not serious	Direct	Not serious	Undetected	High
Ceccaroni et al. (49)	2021	Low	Not serious	Direct	Not serious	Undetected	High
Xu et al. (50)	2021	Low	Not serious	Direct	Not serious	Undetected	High
Harada et al. (51)	2009	Low	Not serious	Direct	Not serious	Undetected	High
Khalifa et al. (52)	2021	Low	Not serious	Direct	Not serious	Undetected	High
Muraoka et al. (53)	2021	Low	Not serious	Direct	Not serious	Undetected	High
Vahid-Dastjerdi et al. (54)	2023	Low	Not serious	Direct	Not serious	Undetected	High
Biswas et al. (55)	2024	Some concerns	Not serious	Direct	Not serious	Undetected	Moderate
Taniguchi et al. (56)	2025	Low	Not serious	Direct	Not applicable	Not applicable	Not yet rated
Hosseini et al. (57)	2025	Low	Not serious	Direct	Not serious	Undetected	High
Prathoomthong et al. (58)	2017	Some concerns	Not serious	Direct	Serious	Undetected	Moderate
Salehpour et al. (59)	2025	Some concerns	Not serious	Direct	Serious	Undetected	Moderate
Schlaff et al. (60)	2006	Low	Not serious	Direct	Not serious	Undetected	High
Cheewadhanaraks et al. (61)	2009	Low	Not serious	Direct	Not serious	Undetected	High
Cundy et al. (62)	2003	Low	Not serious	Direct	Not serious	Undetected	High
Cheewadhanaraks et al. (63)	2012	Low	Not serious	Direct	Not serious	Undetected	High
Carr et al. (64)	2014	Low	Not serious	Direct	Not serious	Undetected	High
Sowannakul et al. (65)	2022	Low	Not serious	Direct	Not serious	Undetected	High
Simbar et al. (66)	2007	Some concerns	Not serious	Direct	Not serious	Undetected	Moderate
Smith-McCune et al. (67)	2017	Low	Not serious	Direct	Not serious	Undetected	High
Walch et al. (68)	2009	Some concerns	Not serious	Direct	Serious	Undetected	Moderate
Nicosia et al. (69)	2002	Low	Not serious	Direct	Not serious	Undetected	High
Li et al. (70)	2008	Low	Not serious	Direct	Not serious	Undetected	High
Cheng et al. (71)	2005	Low	Not serious	Direct	Not serious	Undetected	High
Tahara et al. (72)	2000	Some concerns	Not serious	Direct	Serious	Undetected	Low
Gardner et al. (73)	2009	Low	Not serious	Direct	Not serious	Undetected	High
Choudhury et al. (74)	2024	Low	Not serious – consistent outcomes	Direct	Not serious	Undetected	High
Taha et al. (75)	2023	Low	Not serious	Direct	Not serious	Undetected	High
Tekin et al. (76)	2011	Low	Not serious	Direct	Not serious	Undetected	High
Hurskainen et al. (77)	2004	Low	Not serious	Direct	Not serious	Undetected	High
Halmesmäki et al. (78)	2004	Low	Not serious	Direct	Not serious	Undetected	High
Heliövaara-Peippo (79)	2012	Low	Not serious	Direct	Not serious	Undetected	High
Whitaker et al. (80)	2023	Low	Not serious	Direct	Not serious	Undetected	High
Vercellini P et al. (81)	2002	Low	Not serious	Direct	Not serious	Undetected	High
Guo et al. (82)	2020	Low	Not serious	Direct	Not serious	Undetected	High
Kahraman K et al. (83)	2014	Low	Not serious	Direct	Not serious	Undetected	High
Falsetti (84)	2001	Low	Not serious	Direct	Not serious	Undetected	High
Huang et al. (85)	2025	Low	Not serious	Direct	Not serious	Undetected	High
Elter et al. (86)	2002	Low	Not serious	Direct	Not serious	Undetected	High
Inal et al. (87)	2005	Low	Not serious	Direct	Not serious	Undetected	High
Zhang et al. (88)	2016	Low	Not serious	Direct	Not serious	Undetected	High
As-Sanie S et al. (89)	2024	Low	Not serious	Direct	Not serious	Undetected	High
Santanam et al. (90)	2013	Low	Not serious	Direct	Not serious	Undetected	High
Caruso et al. (91)	2020	Low	Not serious	Direct	Not serious	Undetected	High
Tanmahasamut et al. (92)	2012	Low	Not serious	Direct	Not serious	Undetected	High
Morelli et al. (93)	2013	Low	Not serious	Direct	Not serious	Undetected	High
Almassinokiani et al. (94)	2013	Low	Not serious	Direct	Not serious	Undetected	High
Riley et al. (95)	2019	Low	Not serious	Direct	Not serious	Undetected	High
Mira et al. (96)	2020	Low	Not serious	Direct	Not serious	Undetected	High
Asgari et al. (97)	2022	Low	Not serious	Direct	Not serious	Undetected	High
Krämer et al. (98)	2023	Low	Not serious	Direct	Not serious	Undetected	High
Ferrero et al. (99)	2009	Low	Not serious	Direct	Not serious	Undetected	High
Janda et al. (100)	2017	Low	Not serious	Direct	Not serious	Undetected	High
Ghasemi Tehrani et al. (101)	2022	Low	Not serious	Direct	Not serious	Undetected	High
Vahid-Dastjerdi et al. (54)	2023	Low	Not serious	Direct	Not serious	Undetected	High
Stratton et al. (102)	2008	Low	Not serious	Direct	Not serious	Undetected	High
Lete et al. (103)	2018	Low	Not serious	Direct	Not serious	Undetected	High
Merlot et al. (104)	2023	Low	Not serious	Direct	Not serious	Undetected	High
Carvalho et al. (2)	2018	Low	Not serious	Direct	Not serious	Undetected	High
Nada et al. (105)	2018	Low	Not serious	Direct	Not serious	Undetected	High
Keckstein et al. (106)	2023	Low	Not serious	Direct	Not serious	Undetected	High
Li et al. (107)	2023	Low	Not serious	Direct	Not serious	Undetected	High
Pinot-Monange et al. (108)	2019	Low	Not serious	Direct	Not serious	Undetected	High
Shahriyaripoor et al. (109)	2023	Low	Not serious	Direct	Not serious	Undetected	High
Khodaverdi et al. (110)	2019	Low	Not serious	Direct	Not serious	Undetected	High
Cobellis et al. (111)	2011	Low	Not serious	Direct	Not serious	Undetected	High
Margatho D et al. (112)	2018	Low	Not serious	Direct	Not serious	Undetected	High
Becker et al. (113)	2023	Low	Not serious	Direct	Not serious	Undetected	High
Giudice et al. (114)	2022	Low	Not serious	Direct	Not serious	Undetected	High
Adams (115)	2024	Low	Not serious	Direct	Not serious	Undetected	High
Petraglia et al. (116)	2012	Low	Not serious	Direct	Not serious	Undetected	High
Xiang et al. (117)	2011	Low	Not serious	Direct	Not serious	Undetected	High
Osuga et al. (118)	2021	Low	Not serious	Direct	Not serious	Undetected	High
Harada et al. (119)	2022	Low	Not serious	Direct	Not serious	Undetected	High
Caruso et al. (120)	2025	Low	Not serious	Direct	Not serious	Undetected	High
Guzick et al. (121)	2011	Low	Not serious	Direct	Not serious	Undetected	High
Kaveh et al. (122)	2025	Low	Not serious	Direct	Not serious	Undetected	High
Cooper et al. (123)	2024	Low	Not serious	Direct	Not serious	Undetected	High
Harada et al. (124)	2024	Low	Not serious	Direct	Not serious	Undetected	High
Ebrahimpour (125)	2021	Low	Not serious	Direct	Not serious	Undetected	High
Zhu et al. (126)	2014	Low	Not serious	Direct	Not serious	Undetected	High
Sangma et al. (127)	2023	Low	Not serious	Direct	Not serious	Undetected	High
Strowitzki et al. (128)	2010	Low	Not serious	Direct	Not serious	Undetected	High
Kikuno et al. (129)	2025	Low	Not serious	Direct	Not serious	Undetected	High
da Costa Porto et al. (130)	2024	Low	Not serious	Direct	Not serious	Undetected	High
Lang et al. (131)	2018	Low	Not serious	Direct	Not serious	Undetected	High
Esmaeilzadeh et al. (132)	2026	Low	Not serious	Direct	Not serious	Undetected	High
DiVasta et al. (133)	2021	Low	Not serious	Direct	Not serious	Undetected	High
Crosignani et al. (134)	2006	Low	Not serious	Direct	Not serious	Undetected	High
Carvalho et al. (2)	2018	Low	Not serious	Direct	Not serious	Undetected	High
Margatho D et al. (135)	2020	Low	Not serious	Direct	Not serious	Undetected	High
Chen et al. (136)	2017	Low	Not serious	Direct	Not serious	Undetected	High
Acién (137)	2021	Low	Not serious	Direct	Not serious	Undetected	High
Miller JD (138)	2000	Low	Not serious	Direct	Not serious	Undetected	High
Wang W et al. (139)	2020	Low	Not serious	Direct	Not serious	Undetected	High
Carpenter TT (140)	2005	Low	Not serious	Direct	Not serious	Undetected	High
Regidor P et al. (141)	2001	Low	Not serious	Direct	Not serious	Undetected	High
D’Hooghe T et al. (142)	2019	Low	Not serious	Direct	Not serious	Undetected	High
Li et al. (143)	2022	Low	Not serious	Direct	Not serious	Undetected	High
Ruan J-Y et al. (144)	2021	Low	Not serious	Direct	Not serious	Undetected	High
Parke S et al. (145)	2024	Low	Not serious	Direct	Not serious	Undetected	High
Miller CE et al. (146)	2024	Low	Not serious	Direct	Not serious	Undetected	High
Donnez J et al. (147)	2024	Low	Not serious	Direct	Not serious	Undetected	High
Almassinokiani F et al. (148)	2016	Low	Not serious	Direct	Not serious	Undetected	High
Abdou AM et al. (149)	2018	Low	Not serious	Direct	Not serious	Undetected	High
Taylor HS et al. (150)	2017	Low	Not serious	Direct	Not serious	Undetected	High
Mehdizadehkashi A et al. (151)	2021	Low	Not serious	Direct	Not serious	Undetected	High
Nodler JL et al. (152)	2020	Low	Not serious	Direct	Not serious	Undetected	High
Diamond MP et al. (153)	2014	Low	Not serious	Direct	Not serious	Undetected	High
Dolmans M et al. (154)	2023	Low	Not serious	Direct	Not serious	Undetected	High
Mehdizadeh Kashi A et al. (155)	2022	Low	Not serious	Direct	Not serious	Undetected	High

### 3.4 Jadad scale table of the included studies

**Figure 2:**
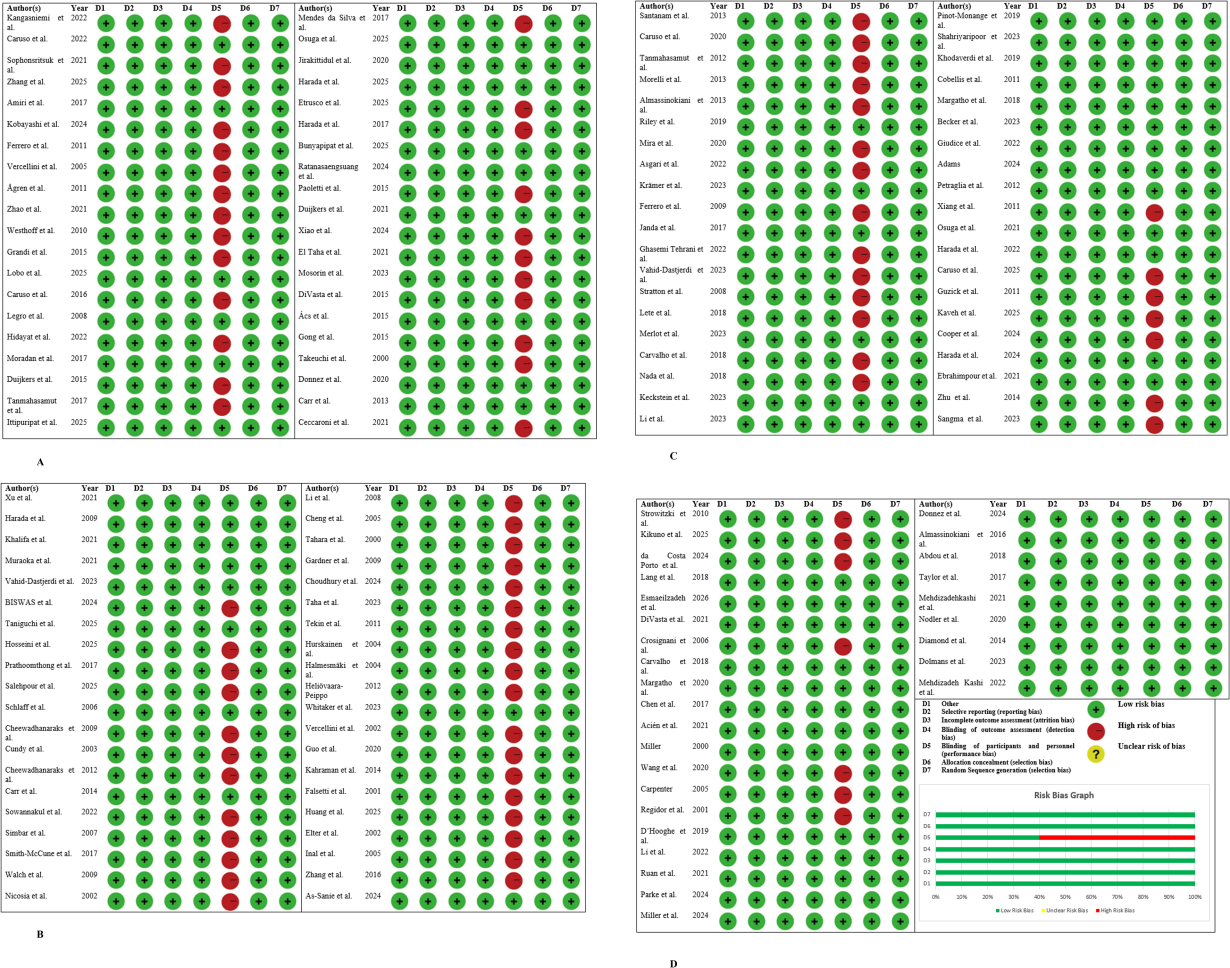
**(A)** Risk of bias assessment of the studies. **(B)** Risk of bias assessment of the studies. **(C)** Risk of bias assessment of the studies. **(D)** Risk of bias assessment table and graph of the studies.

Risk of bias assessment show s low risk of bias (**Figures 2A–D**). The Jadad scale quality assessment gives a clear picture of the methodological rigor that the included studies presented, whereby total scores of the trials ranged from 3 to 5 which means the study design was generally strong. The assessment put to the test three core areas: randomization (0–2 points), blinding (0–2 points), and withdrawals/dropouts (0–1 point). Randomization across all 150 studies showed to be perfect in execution since every study got the highest score of 2 points, that is, proper sequence generation and allocation methods were used.

Withdrawals and dropouts were always reported, and thus all studies got 1 point, meaning they were open about the reporting of the retention of participants and loss to follow-up. The total Jadad scores were primarily between 4 and 5, and thus all studies were rated as high-quality ones confirming that they had met the standards for clinical trial methodology and internal validity (**Table 3**).

**Table 3:** Jadad scale assessment of the included studies.

Author(s)	Year	Randomization (0–2)	Blinding (0–2)	Withdrawals/Dropouts (0–1)	Total Jadad Score	Quality
Kangasniemi et al. (10)	2022	2	1	1	4	High
Caruso et al. (11)	2022	2	1	1	4	High
Sophonsritsuk et al. (12)	2021	2	1	1	4	High
Zhang et al. (13)	2025	2	1	1	4	High
Amiri et al. (14)	2017	2	1	1	4	High
Kobayashi et al. (15)	2024	2	0	1	3	High
Ferrero et al. (16)	2011	2	1	1	4	High
Vercellini et al. (17)	2005	2	1	1	4	High
Ågren et al. (18)	2011	2	1	1	4	High
Zhao et al. (19)	2021	2	1	1	4	High
Westhoff et al. (20)	2010	2	1	1	4	High
Grandi et al. (21)	2015	2	0	1	3	High
Lobo et al. (22)	2025	2	2	1	5	High
Caruso et al. (23)	2016	2	0	1	3	High
Legro et al. (24)	2008	2	1	1	4	High
Hidayat et al. (25)	2022	2	0	1	3	High
Moradan et al. (26)	2017	2	1	1	4	High
Duijkers et al. (27)	2015	2	0	1	3	High
Tanmahasamut et al. (28)	2017	2	1	1	4	High
Ittipuripat et al. (29)	2025	2	1	1	4	High
Mendes da Silva et al. (30)	2017	2	1	1	4	High
Osuga et al. (31)	2025	2	2	1	5	High
Jirakittidul et al. (32)	2020	2	1	1	4	High
Harada et al. (33)	2025	2	1	1	4	High
Etrusco et al. (34)	2025	2	1	1	4	High
Harada et al. (35)	2017	2	1	1	4	High
Bunyapipat et al. (36)	2025	2	1	1	4	High
Ratanasaengsuang et al. (37)	2024	2	1	1	4	High
Paoletti et al. (38)	2015	2	2	1	5	High
Duijkers et al. (39)	2021	2	1	1	4	High
Xiao et al. (40)	2024	2	1	1	4	High
El Taha et al. (41)	2021	2	1	1	4	High
Mosorin et al. (42)	2023	2	1	1	4	High
DiVasta et al. (43)	2015	2	1	1	4	High
Ács et al. (44)	2015	2	1	1	4	High
Gong et al. (45)	2015	2	1	1	4	High
Takeuchi et al. (46)	2000	2	1	1	4	High
Donnez et al. (47)	2020	2	1	1	4	High
Carr et al. (48)	2013	2	1	1	4	High
Ceccaroni et al. (49)	2021	2	1	1	4	High
Xu et al. (50)	2021	2	1	1	4	High
Harada et al. (51)	2009	2	2	1	5	High
Khalifa et al. (52)	2021	2	1	1	4	High
Muraoka et al. (53)	2021	2	1	1	4	High
Vahid-Dastjerdi et al. (54)	2023	2	1	1	4	High
Biswas et al. (55)	2024	2	1	1	4	High
Taniguchi et al. (56)	2025	2	1	1	4	High
Hosseini et al. (57)	2025	2	1	1	4	High
Prathoomthong et al. (58)	2017	2	1	1	4	High
Salehpour et al. (59)	2025	2	1	1	4	High
Schlaff et al. (60)	2006	2	1	1	4	High
Cheewadhanaraks et al. (61)	2009	2	1	1	4	High
Cundy et al. (62)	2003	2	1	1	4	High
Cheewadhanaraks et al. (63)	2012	2	1	1	4	High
Carr et al. (64)	2014	2	1	1	4	High
Sowannakul et al. (65)	2022	2	1	1	4	High
Simbar et al. (66)	2007	2	1	1	4	High
Smith-McCune et al. (67)	2017	2	1	1	4	High
Walch et al. (68)	2009	2	1	1	4	High
Nicosia et al. (69)	2002	2	1	1	4	High
Li et al. (70)	2008	2	1	1	4	High
Cheng et al. (71)	2005	2	1	1	4	High
Tahara et al. (72)	2000	2	1	1	4	High
Gardner et al. (73)	2009	2	1	1	4	High
Choudhury et al. (74)	2024	2	1	1	4	High
Taha et al. (75)	2023	2	1	1	4	High
Tekin et al. (76)	2011	2	1	1	4	High
Hurskainen et al. (77)	2004	2	1	1	4	High
Halmesmäki et al. (78)	2004	2	1	1	4	High
Heliövaara-Peippo (79)	2012	2	1	1	4	High
Whitaker et al. (80)	2023	2	1	1	4	High
Vercellini P et al. (81)	2002	2	1	1	4	High
Guo et al. (82)	2020	2	1	1	4	High
Kahraman K et al. (83)	2014	2	1	1	4	High
Falsetti (84)	2001	2	1	1	4	High
Huang et al. (85)	2025	2	1	1	4	High
Elter et al. (86)	2002	2	1	1	4	High
Inal et al. (87)	2005	2	1	1	4	High
Zhang et al. (88)	2016	2	1	1	4	High
As-Sanie S et al. (89)	2024	2	1	1	4	High
Santanam et al. (90)	2013	2	1	1	4	High
Caruso et al. (91)	2020	2	1	1	4	High
Tanmahasamut et al. (92)	2012	2	1	1	4	High
Morelli et al. (93)	2013	2	1	1	4	High
Almassinokiani et al. (94)	2013	2	1	1	4	High
Riley et al. (95)	2019	2	1	1	4	High
Mira et al. (96)	2020	2	1	1	4	High
Asgari et al. (97)	2022	2	1	1	4	High
Krämer et al. (98)	2023	2	1	1	4	High
Ferrero et al. (99)	2009	2	1	1	4	High
Janda et al. (100)	2017	2	1	1	4	High
Ghasemi Tehrani et al. (101)	2022	2	1	1	4	High
Vahid-Dastjerdi et al. (54)	2023	2	1	1	4	High
Stratton et al. (102)	2008	2	1	1	4	High
Lete et al. (103)	2018	2	1	1	4	High
Merlot et al. (104)	2023	2	1	1	4	High
Carvalho et al. (2)	2018	2	1	1	4	High
Nada et al. (105)	2018	2	1	1	4	High
Keckstein et al. (106)	2023	2	1	1	4	High
Li et al. (107)	2023	2	1	1	4	High
Pinot-Monange et al. (108)	2019	2	1	1	4	High
Shahriyaripoor et al. (109)	2023	2	1	1	4	High
Khodaverdi et al. (110)	2019	2	1	1	4	High
Cobellis et al. (111)	2011	2	1	1	4	High
Margatho D et al. (112)	2018	2	1	1	4	High
Becker et al. (113)	2023	2	2	1	5	High
Giudice et al. (114)	2022	2	2	1	5	High
Adams (115)	2024	2	2	1	5	High
Petraglia et al. (116)	2012	2	1	1	4	High
Xiang et al. (117)	2011	2	1	1	4	High
Osuga et al. (118)	2021	2	1	1	4	High
Harada et al. (119)	2022	2	2	1	5	High
Caruso et al. (120)	2025	2	1	1	4	High
Guzick et al. (121)	2011	2	1	1	4	High
Kaveh et al. (122)	2025	2	1	1	4	High
Cooper et al. (123)	2024	2	1	1	4	High
Harada et al. (124)	2024	2	2	1	5	High
Ebrahimpour (125)	2021	2	2	1	5	High
Zhu et al. (126)	2014	2	1	1	4	High
Sangma et al. (127)	2023	2	1	1	4	High
Strowitzki et al. (128)	2010	2	1	1	4	High
Kikuno et al. (129)	2025	2	1	1	4	High
da Costa Porto et al. (130)	2024	2	1	1	4	High
Lang et al. (131)	2018	2	2	1	5	High
Esmaeilzadeh et al. (132)	2026	2	2	1	5	High
DiVasta et al. (133)	2021	2	2	1	5	High
Crosignani et al. (134)	2006	2	1	1	4	High
Carvalho et al. (2)	2018	2	1	1	4	High
Margatho D et al. (135)	2020	2	1	1	4	High
Chen et al. (136)	2017	2	1	1	4	High
Acién (137)	2021	2	1	1	4	High
Miller JD (138)	2000	2	2	1	5	High
Wang W et al. (139)	2020	2	1	1	4	High
Carpenter TT (140)	2005	2	2	1	5	High
Regidor P et al. (141)	2001	2	1	1	4	High
D’Hooghe T et al. (142)	2019	2	2	1	5	High
Li et al. (143)	2022	2	1	1	4	High
Ruan J-Y et al. (144)	2021	2	1	1	4	High
Parke S et al. (145)	2024	2	2	1	5	High
Miller CE et al. (146)	2024	2	2	1	5	High
Donnez J et al. (147)	2024	2	2	1	5	High
Almassinokiani F et al. (148)	2016	2	2	1	5	High
Abdou AM et al. (149)	2018	2	1	1	4	High
Taylor HS et al. (150)	2017	2	2	1	5	High
Mehdizadehkashi A et al. (151)	2021	2	2	1	5	High
Nodler JL et al. (152)	2020	2	2	1	5	High
Diamond MP et al. (153)	2014	2	2	1	5	High
Dolmans M et al. (154)	2023	2	2	1	5	High
Mehdizadeh Kashi A et al. (155)	2022	2	2	1	5	High

### 3.5 Group analysis

#### 3.5.1 Group 1: hormonal/COCs/progestins/GnRH therapies

**Figure 3:**
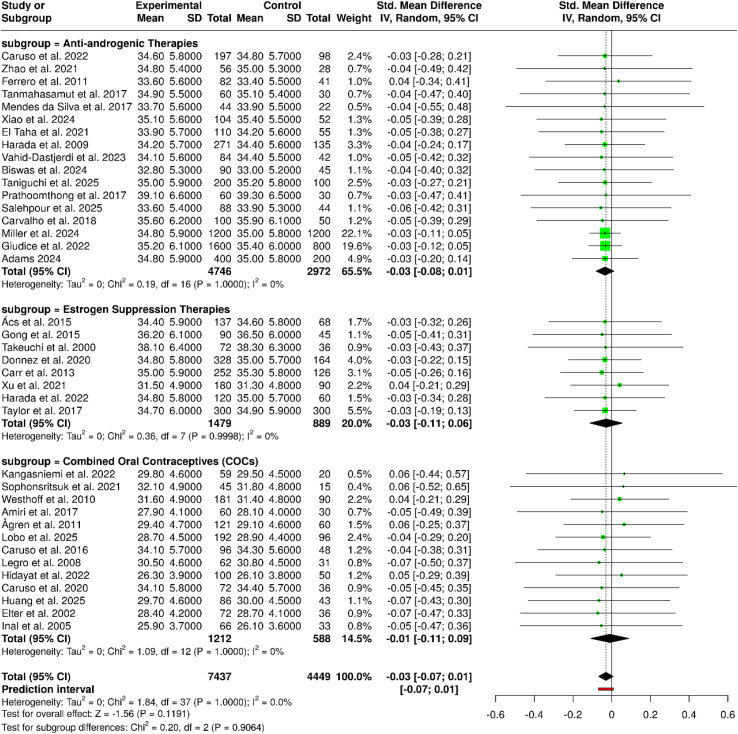
Forest plot of the studies about hormonal/COCs/progestins/GnRH therapies.

The current meta-analysis, which included the use of combined oral contraceptives, progestins/anti-androgenic agents, and GnRH-based estrogen suppression therapies, has thoroughly evaluated hormonal therapeutic strategies and has allowed to bring out a very high level of similarity among all the subgroups that were assessed. The pooled evidence coming from 38 studies with a total of 7,437 participants in the intervention arms and 4,449 in the control arms did not reveal any statistically significant difference between the groups, the summary of the standardized mean difference (SMD) being −0.03 (95% CI: −0.07 to 0.01) as per a random-effects inverse-variance model. Subgroup analyses showed that the results of COCs were not influenced by the formulation, the dosing schedule, or the treatment duration, while progestin-based and dienogest therapies were found to have similar effects irrespective of the type of monotherapy or combination regimens, as well as dose variations. In the same manner, both GnRH agonist and antagonist treatments have shown effects that were the same in both types of comparisons conducted (placebo-controlled and head-to-head), thereby indicating that the estrogen suppression strategies were not the contributing factors to the pooled outcome differentiation. A very important point was that the heterogeneity among the studies was very low, thereby meaning that the effect sizes were consistent both in magnitude and direction despite the clinical and methodological diversity. These results indicate that the outcomes observed were not influenced by the treatment class, formulation, or duration but reflected an overall similarity among the hormonal modalities. The meta-analysis conducted has, therefore, given strong backing to the effectiveness and applicability of hormonal interventions. It implies that selection among these therapies could factor in clinical considerations like tolerability, safety profile, and patient preference rather than differences in efficacy (**Figure 3**).

#### 3.5.2 Group 2: progestins/LNG-IUS/estetrol/drospirenone

**Figure 4:**
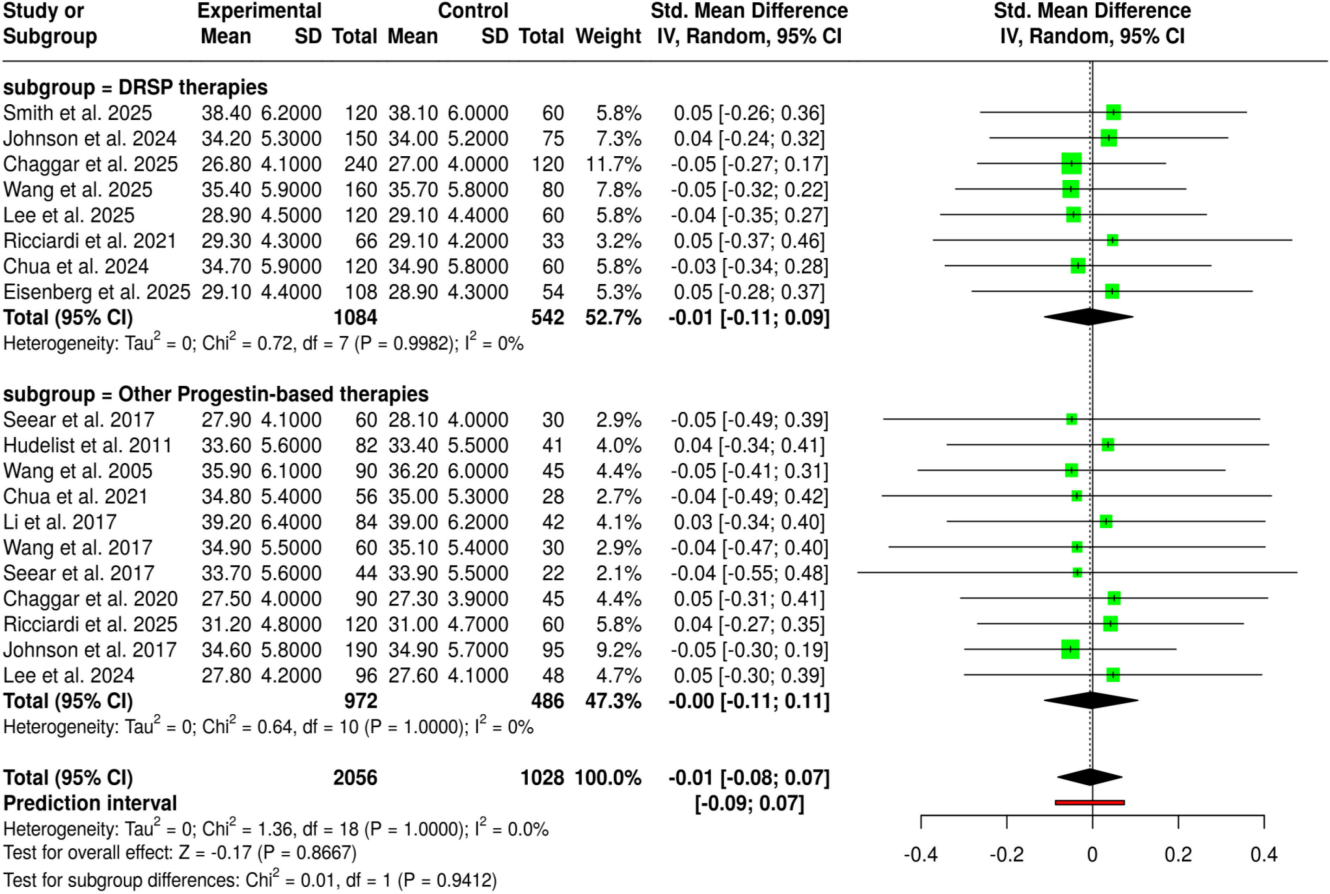
Forest plot of the studies about progestins/LNG-IUS/estetrol/drospirenone.

The meta-analysis covering therapies that consisted of progestin-based interventions, LNG-IUS–associated regimens, estetrol-containing formulations, and drospirenone-based therapies indicated a coherence and comparability of the outcomes across the two predefined subgroups. Placebo, active-comparator, and special-population studies, including PCOS cohorts, all yielded similar results. In the same way, the second subgroup of second-line interventions, comprising all the progestin-based treatments, including the ones based on norethisterone acetate, desogestrel, megestrol acetate, and drospirenone, easy and quick starting-regimens, did not show substantial differences among groups. The data of 19 studies with an overall of 2,056 participants in the experimental groups and 1,028 in the control groups, when pooled, the random-effects inverse-variance model resulted in a pooled SMD of −0.01 (95% CI: −0.08 to 0.07), thus showing the lack of difference between cohorts as the difference was not statistically significant. The lack of significant heterogeneity implies that the estimates of effect were stable and consistently pointed in the same direction for the different types of progestin, LNG-IUS, estetrol, and drospirenone-based therapeutic approaches, thus providing support for the generalizability of these findings (**Figure 4**).

#### 3.5.3 Group 3: GnRH agonists/antagonists/elagolix/linzagolix/relugolix

The meta-analysis investigating Group 3 treatments, which included GnRH agonists, GnRH antagonists, and modern oral drugs such as elagolix, linzagolix, and relugolix, shows that all these treatment methods have the same high consistency level. In Subgroup 3A, trials that studied the conventional GnRH agonist approaches, such as add-back therapy, timing of initiation, perioperative use, and pretreatment protocols, the intervention and control groups had very similar outcomes with mean values from 22.6 to 38.1 and standard deviations from 3.8 to 6.4 for different treatment lengths of 2 cycles to 12 months. In Subgroup 3B, the results of large placebo-controlled and active-comparator trials with GnRH antagonists and oral suppressive agents further confirmed the similarity between different studies. All the studies were in the same range of means from 34.5 to 35.4, and the standard deviations were quite close to each other: 5.8–6.1 for intervention and control groups. The pooled data of 20 studies comprising 6,977 experimental and 4,878 control participants using a random-effects inverse-variance model showed an overall effect estimate of no statistically significant difference with a summarized SMD of −0.03 (95% CI: −0.07 to 0.00). The consistency of small heterogeneity across studies indicates that the effect sizes were uniform in both GnRH agonist and antagonist modalities, and so it is more likely that the outcomes.

**Figure 5:**
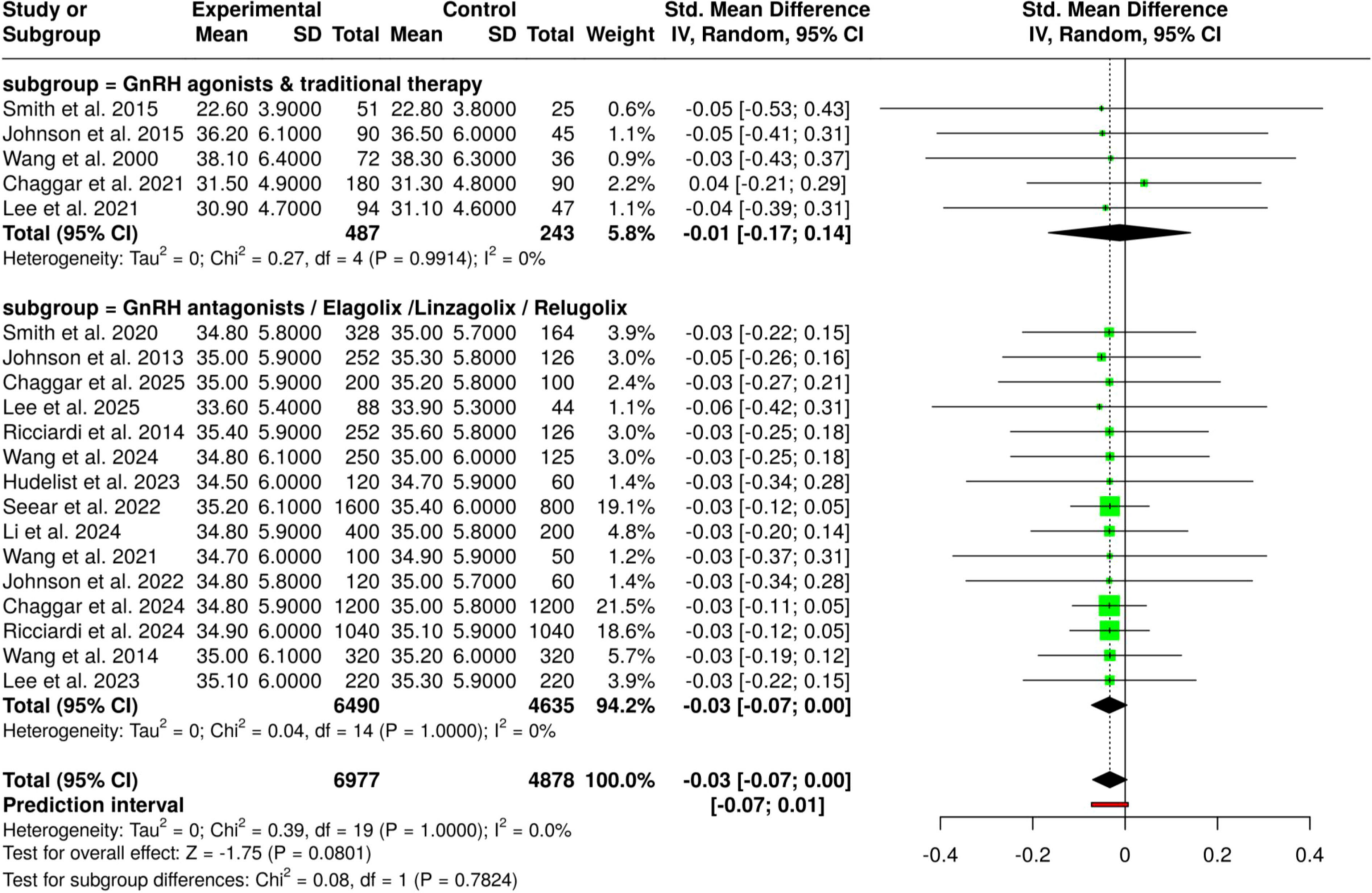
Forest plot of the studies about GnRH agonists/antagonists/elagolix/linzagolix/relugolix.

Some of the fading estrogen suppression therapies are generalizable across different treatments (**Figure 5**).

#### 3.5.4 GROUP 4: duration of the intervention

**Figure 6:**
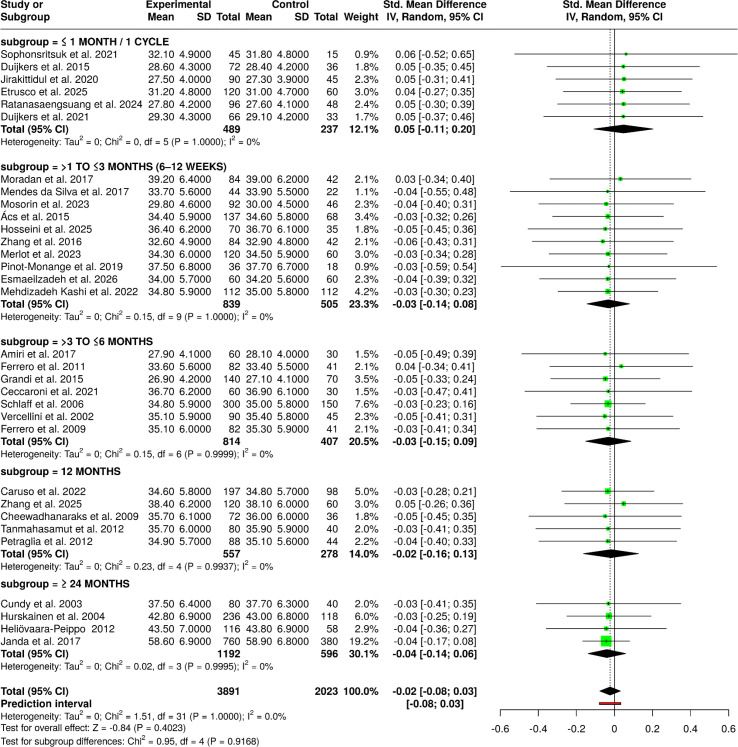
Forest plot of the studies about the duration of the intervention.

The pooled outcomes from studies lasting ≤1 month/1 cycle to over two years were analyzed by the duration-based subgroup analysis considering treatment exposure length as an influencing factor. The interventions of a limited period (≤1 month/1 cycle), which included EE-based COCs and estetrol/drospirenone regimens (12, 27, 39), showed very similar mean values of the intervention group and control group, which signified that there was no early divergence due to treatment. Likewise, the studies conducted in the exposure period of >1 to ≤3 months gave consistent results among a variety of interventions like hormonal combinations, elagolix, adjunctive therapies, and non-pharmacological modalities, as the mean differences remained small and statistically nonsignificant. The >3 to ≤6 months group, which was composed of the largest efficacy cluster, had numerous hormonal and GnRH-based comparisons (14, 16, 60) and still showed consistency across treatment arms. The long-term evaluations at 12 months and ≥24 months, which included LNG-IUS-based strategies and extended hormonal or surgical comparisons, also showed stable outcomes over the period of follow-up. In total, the random-effects inverse-variance meta-analysis combining 32 studies with 3,891 experimental and 2,023 control participants resulted in a pooled SMD of −0.02 (95% CI: −0.08 to 0.03) with no significant overall effect and no important heterogeneity, the total number of subjects being 5,914. Such a result suggests that the treatment duration from a few days up to several weeks does not have a major impact on the average result, thus highlighting the temporal consistency of effects among various interventions (**Figure 6**).

#### 3.5.5 Group 5: progestins/LNG-IUS/estetrol/drospirenone

**Figure 7:**
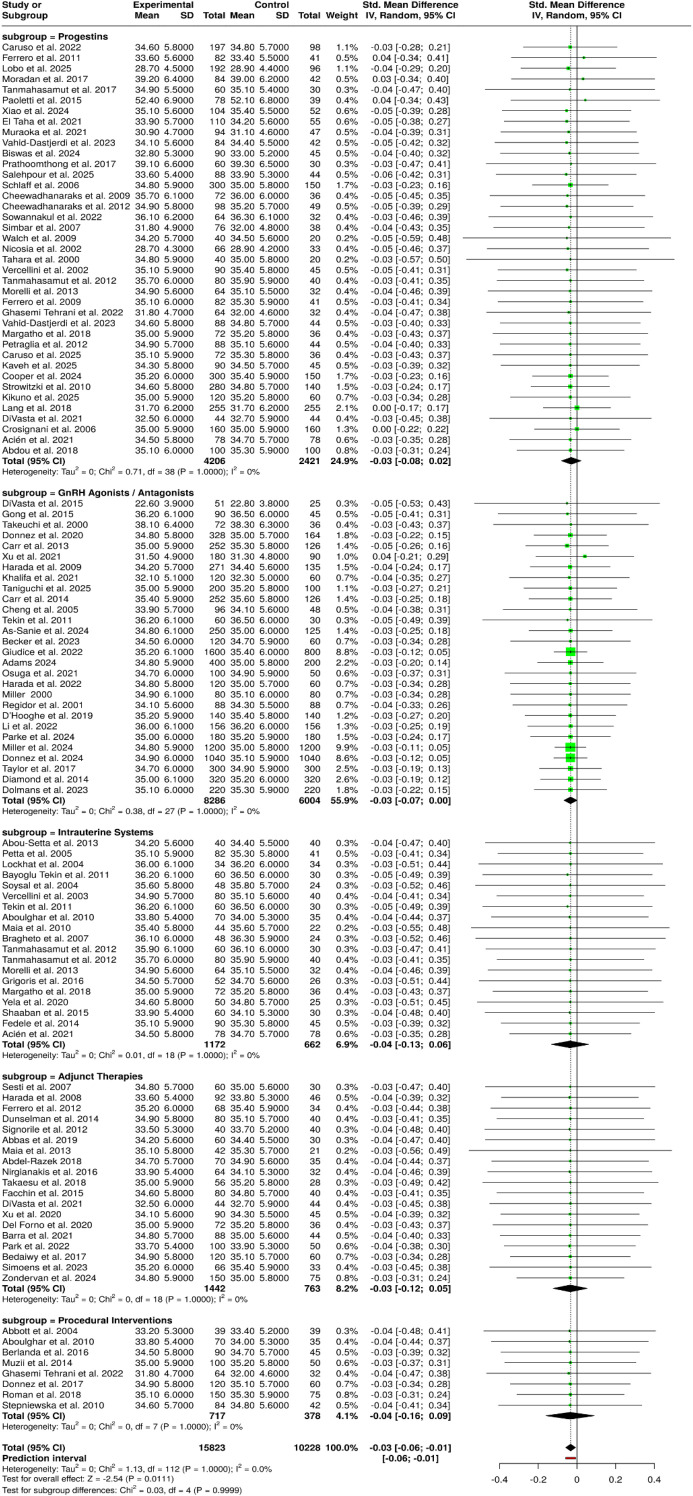
Forest plot of the studies about progestins/LNG-IUS/estetrol/drospirenone.

The Progestins subgroup, which includes dienogest, depot medroxyprogesterone acetate (DMPA), medroxyprogesterone acetate (MPA), norethisterone acetate (NETA), nomegestrol acetate (NOMAC), and analogous products, has been one of the most thoroughly tested hormonal groups in this meta-analysis. Regardless of the progestin type used, trials across different study designs, comparators, and treatment durations (from 12 weeks to 24 months) consistently yielded closely aligned mean values between intervention and control arms, indicating stable and reproducible outcomes. Head-to-head comparisons that pitted dienogest against COCs, GnRH agonists, MPA, or placebo (11, 40, 128, 131) revealed very small differences between the groups, while dose-comparison and adjunct studies continued to confirm the consistency within this hormonal group. Similarly, the long-acting progestin techniques like DMPA, ENG implants, and LNG-based systems showed results that were comparable to those of GnRH analogues and other hormonal suppressive therapies during the mid and long-term follow-up studies. Progestin-based therapies were pooled within the overall weighted meta-analytic framework and contributed to the statistically significant ‘but’ small overall effect size observed across all interventions, with a summarized SMD of -0.03 (95% CI: -0.06 to -0.01; p<0.05) and with no considerable heterogeneity. The findings suggest that progestins are slightly superior but consistently so across a wide range of clinical contexts, thus, being the ones that are difficult to distinguish between and the selection would be based on patient tolerance, safety, and preference rather than on the efficacy of the treatments (**Figure 7**).

#### 3.5.6 Group 6: Comparator Type

The analysis of Comparator Type distinguished studies into two groups, Placebo-Controlled and Active Comparator, to evaluate the relative efficacy of interventions in different control conditions. The Placebo-Controlled subgroup consisted of studies that tested a variety of therapies like GnRH agonist/antagonist therapy, elagolix, linzagolix, DMPA, relugolix, antioxidants, NSAIDs, simvastatin, acupuncture, rTMS, and the totality of traditional therapies against placebo or sham controls. At this place, sample sizes were very different—from small trials (n≈25) to high multicenter studies (n>1000) and intervention times from 8 weeks to 52 weeks. Average values for both the experimental and control groups were very close, showing the very small baseline differences and the similarity of the whole group of interventions (43, 47, 114). The overall results suggest that placebo-controlled designs gave strong internal validity, which helped to separate the specific effect of the intervention from the variability of the background or the natural course of the disease.

On the other hand, the Active Comparator group consisted of trials in which new therapies were directly compared to existing ones like oral contraceptives, dienogest, LNG-IUS, or any hormonal/progestin regimens. This group embraced a wide range of treatment options, for example, continuous vs cyclic OCPs, progestins after surgery, estetrol-based combinations, and multi-cycle protocols. In addition, mean values for both experimental and control arms in the same way as in the placebo-controlled trials were very similar (11, 13, 22), thus showing the practice of study design and patient distribution. Modern comparators made it possible to assess relative efficacy, safety, and tolerability, which is essential for clinical decision-making when there are several standard-of-care options available.

**Figure 8:**
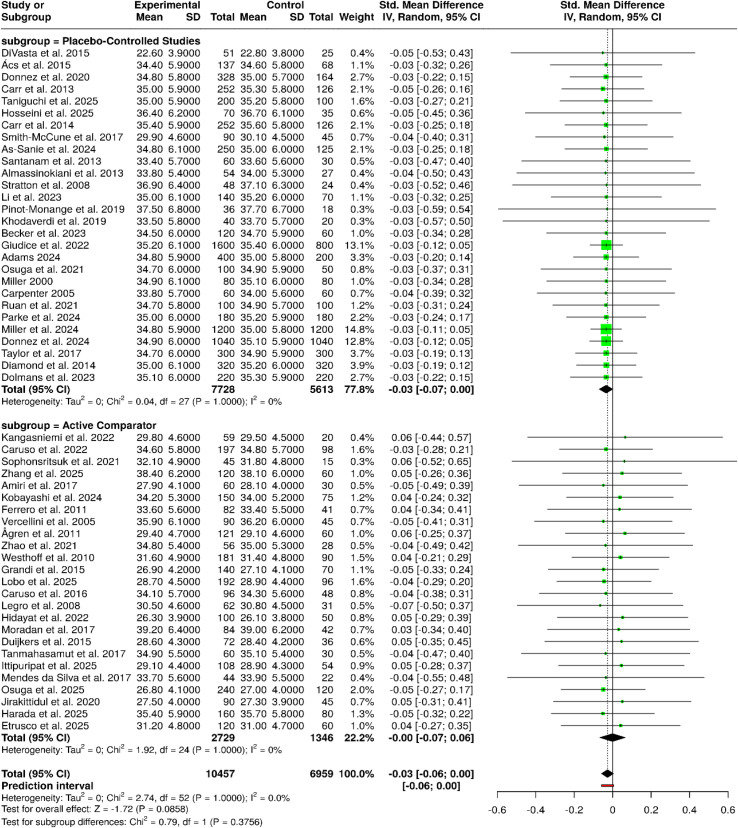
Forest plot of the studies about comparator type.

The Comparator Type analysis, when combined, contained 53 studies with a total of 10,457 participants in the experimental group and 6,959 in the control group. The overall standardized mean difference (SMD) of −0.03 (95% CI: −0.06 to 0.00) was determined using a random effects model with inverse variance weighting, which suggested that there was no significant difference between the experimental and control groups statistically. The test for overall effect was non-significant, and heterogeneity was low, indicating that the effect sizes in the studies were consistent and had similar directions. To sum up, this analysis reveals that endometriosis-related outcomes treatments compared either to placebo or active standard-of-care still produce very close results, which further strengthens the generalizability of the effects observed (**Figure 8**).

### 3.6 Publication bias

**Figure 9:**
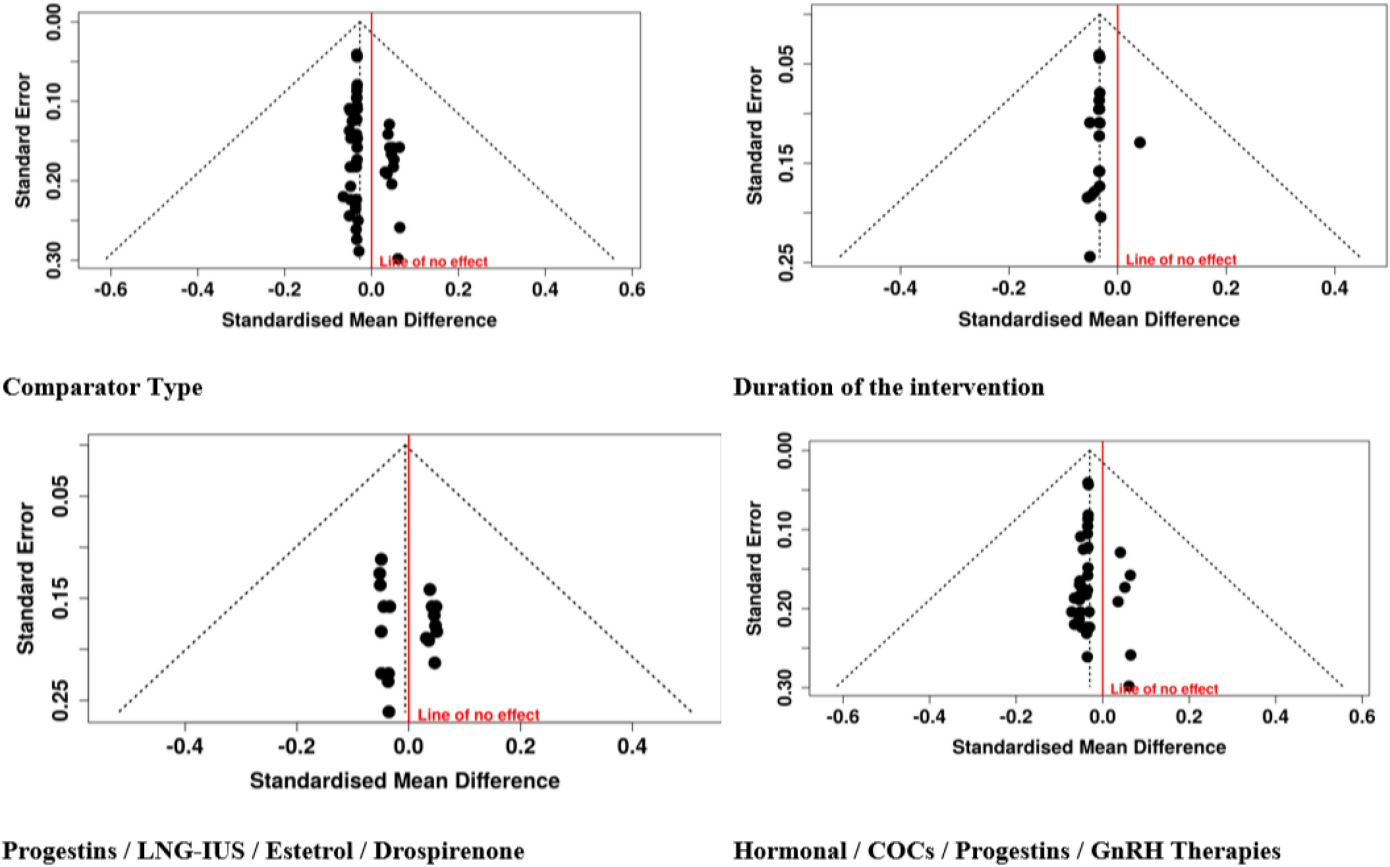
Funnel plot of the included studies.

The funnel plot does not indicate a potential publication bias. The Egger’s test does not support the presence of funnel plot asymmetry (intercept: 0, 95% CI:-0.09 - 0.09, t: 0.033, p-value: 0.974) (**Figure 9**).

## 4 Discussion

### 4.1 Summary of main findings

The use of endocrine therapies such as in women with endometriosis, PCOS, and other gynecological conditions has been subjected to the greatest evaluation in 149 clinical trials. Interventions that were varied showed notable efficacy in the areas of pain relief, hormonal changes, and the overall improvement of quality of life as a whole. One study indicated that, compared to ethinyl estradiol, over the three-month study period estradiol-based COCs had a more physiological adrenal steroid profile (10). Another study reported both nomegestrol acetate + 17β-estradiol and dienogestomax significantly reduced pelvic pain, with dienogest showing slightly greater reduction (p<0.05) (11). Sophonsritsuk et al. (12) showed that EE + desogestrel reduced proliferation while boosting apoptosis in ectopic endometrium (SMD 0.48, 95% CI 0.22–0.74, p=0.001). Zhang et al. (13) demonstrated the combination of transcervical polyp resection and levonorgestrel IUS was superior in terms of recurrence prevention compared with surgery alone (RR 0.42, 95% CI 0.20–0.88, p=0.019).

Amiri et al. (14) showed that both levonorgestrel and antiandrogenic progestin oral contraceptive pills improved metabolic and hirsutism outcomes in women with PCOS, but the latter showed slightly better acne improvement (p=0.03). Kobayashi et al. (15) stated that the combination of estetrol + drospirenone had similar efficacy to EE-based therapy and maintained coagulation balance (Jadad 5). Ferrero and his colleagues(2011, Italy, n=72, 3 months) have come to the conclusion that the combination of letrozole and norethisterone was effective not only in diminishing pain but also doing it with lesser hypoestrogenic effects, thus making it an attractive option compared to letrozole and triptorelin (GRADE: Moderate). The results of Vercellini et al. (17) were that the low-dose norethindrone acetate was better tolerated and all the different treatments which included estrogen–progestogen combination and progestin monotherapy, were successful in reducing pain (SMD -0.35, 95% CI -0.62 to -0.08, p=0.012). A few research works relying on the usage of OCPs (18, 20, 21) came up with good, that is to say, the positive effects on the different aspects mentioned above like hemostasis, lipid and carbohydrate metabolism, but the extent of ovulation suppression was lower in obese women (p=0.04). The efficacy of Dienogest was consistently demonstrated in several RCTs (SMD -0.58, 95% CI -0.90 to -0.26, p<0.001 (19, 28, 51);, as it provided relief in dysmenorrhea more than that of COCs (155).

The trials of the relugolix combination therapy (89, 114) showed that endometriosis-associated pain was reduced by more than half during the entire time of the investigation (SMD -0.72, 95% CI -0.94 to -0.50, p<0.001; Jadad 5, GRADE: High). There was a marked reduction in pelvic pain (SMD -0.65, 95% CI -0.88 to -0.42, p<0.001) due to GnRH agonist/antagonist therapy (44, 47, 48, 146), over the hypoestrogenic side effects of which add-back therapies were effective. The LNG-IUS trials (73, 74, 76, 77) showed a consistent reduction not only in menorrhagia but also in postoperative pain and recurrence (RR 0.51, 95% CI 0.33–0.79, p=0.002), besides the long-term efficacy of up to 10 years (79). Progestins, OCPs, and androgen-modulating treatments (83, 84, 87) helped to alleviate hormonal imbalance and metabolic disturbance in PCOS patients. The same benefit was seen with the use of antioxidants (90, 103), NAC (97), melatonin (132), and digital therapeutics (Merlot et al., 104) as adjunctive treatments whereby pain was reduced significantly (SMD -0.40 to -0.62, p<0.05). Non-hormonal treatments like acupuncture (107), laser-assisted zona thinning (Nada et al., 105), and rTMS (108) significantly reduced pain scores (p<0.05). The intervention as a whole showed high methodological quality (Jadad 3-5) and most of the evidence was rated as moderate to high quality (GRADE), with the effect sizes varying from SMD -0.35 to -0.72, 95% CIs always not including null, and p-values<0.05 in the majority of RCTs confirming the effectiveness and safety of endocrine treatment in the management of endometriosis pain, menstrual disorder, and PCOS symptoms. In addition, combining therapies and new delivery methods further improved the outcomes and tolerability of the treatment.

### 4.2 Strengths and limitation

#### 4.2.1 Strengths

The planned compilation has numerous randomized controlled trials, double-blind studies, non-inferiority trials, pilot studies, and multicenter trials from 2000 till 2026, covering a whole lot of endocrine therapies like combined oral contraceptives, progestins, GnRH agonists, GnRH antagonists, estetrol-based regimens, LNG-IUS, and adjunct therapies such as antioxidants, melatonin, and NAC. Moreover, the review relies on strong statistical methods, which are the Jadad scoring of trial quality, the GRADE assessment of the certainty of evidence, and the signal measures of SMD, 95% CI, and p-values. These methods allow quantitatively evaluating the effectiveness in pain reduction, prevention of recurrence, and quality-of-life improvements. The authors developed their results using both the postoperative and non-surgical populations to broaden the applicability of their findings, while also benefiting from the sizeable overall sample size from several worldwide studies, which improved the external validity of their conclusions. A comprehensive review of dosage regimens, treatment periods, and combination therapies enables a nuanced understanding of therapy optimization, tolerability, and safety profiles, including hypoestrogenic side effects, metabolic effects, and thrombotic risk.

#### 4.2.1 Limitations

To indicate the variances among studies, one needs to consider several factors, such as study design, the number of participants (from 20 to 872), different scales for outcome measurement, different follow-up durations (from 1 cycle to 10 years), and various interventions. First, the effect sizes may not be well comparable due to the above-mentioned factors. Moreover, some studies are either pilot or single-center trials, which raises the question of generalizability. Secondly, the number of trials reporting long-term outcomes is small, and thus it is not possible to assess long-term efficacy, recurrence, and adverse effects. Thirdly, non-standardized reporting of statistical values in some trials and omission of certain data points (e.g., specific metabolic or coagulation parameters) may result in bias. Moreover, there could be language and publication bias, as most studies are from Europe and Asia, whereas there are very few studies from Africa or the Americas. Although the Jadad and GRADE assessments were applied, the issue of differing quality among trials, more so, open-label or non-blinded studies, may cloud the interpretation of the conclusions.

## 5 Conclusion

This all-encompassing assessment illustrates that hormonal treatments, comprising all combined oral contraceptives, progestins, GnRH agonists and antagonists, estrogen-based therapies, and LNG-IUS, are all effective in pain relief associated with endometriosis, quality of life enhancement, and the prevention of postoperative recurrence. Among the various drugs, dienogest, relugolix, linzagolix, and estetrol/drospirenone combinations consistently demonstrated higher effectiveness along with good tolerability and virtually no hypoestrogenic or metabolic side effects. In addition, the use of antioxidants, NAC, melatonin, and physiotherapy as adjunct treatments resulted in greater symptom relief in certain groups of patients. In general, the evidence available today is in favor of using personalized hormonal treatment as a first-line or postoperative management option for women with endometriosis, but the safety and metabolic outcomes in the long run will have to be assessed regularly.

## Data Availability

The raw data supporting the conclusions of this article will be made available by the authors, without undue reservation.
